# Neural Correlates of Burnout Syndrome Based on Electroencephalography (EEG)—A Mechanistic Review and Discussion of Burnout Syndrome Cognitive Bias Theory

**DOI:** 10.3390/jcm14155357

**Published:** 2025-07-29

**Authors:** James Chmiel, Agnieszka Malinowska

**Affiliations:** 1 Faculty of Physical Culture and Health, Institute of Physical Culture Sciences, University of Szczecin, Al. Piastów 40B Block 6, 71-065 Szczecin, Poland; 2 Institute of Psychology, University of Szczecin, 71-017 Szczecin, Poland

**Keywords:** burnout, EEG, electroencephalography, electroencephalogram, brain oscillations, QEEG, neurophysiology, neural correlates

## Abstract

**Introduction:** Burnout syndrome, long described as an “occupational phenomenon”, now affects 15–20% of the general workforce and more than 50% of clinicians, teachers, social-care staff and first responders. Its precise nosological standing remains disputed. We conducted a mechanistic review of electroencephalography (EEG) studies to determine whether burnout is accompanied by reproducible brain-function alterations that justify disease-level classification. **Methods:** Following PRISMA-adapted guidelines, two independent reviewers searched PubMed/MEDLINE, Scopus, Google Scholar, Cochrane Library and reference lists (January 1980–May 2025) using combinations of “burnout,” “EEG”, “electroencephalography” and “event-related potential.” Only English-language clinical investigations were eligible. Eighteen studies (n = 2194 participants) met the inclusion criteria. Data were synthesised across three domains: resting-state spectra/connectivity, event-related potentials (ERPs) and longitudinal change. **Results:** Resting EEG consistently showed (i) a 0.4–0.6 Hz slowing of individual-alpha frequency, (ii) 20–35% global alpha-power reduction and (iii) fragmentation of high-alpha (11–13 Hz) fronto-parietal coherence, with stage- and sex-dependent modulation. ERP paradigms revealed a distinctive “alarm-heavy/evaluation-poor” profile; enlarged N2 and ERN components signalled hyper-reactive conflict and error detection, whereas P3b, Pe, reward-P3 and late CNV amplitudes were attenuated by 25–50%, indicating depleted evaluative and preparatory resources. Feedback processing showed intact or heightened FRN but blunted FRP, and affective tasks demonstrated threat-biassed P3a latency shifts alongside dampened VPP/EPN to positive cues. These alterations persisted in longitudinal cohorts yet normalised after recovery, supporting trait-plus-state dynamics. The electrophysiological fingerprint differed from major depression (no frontal-alpha asymmetry, opposite connectivity pattern). **Conclusions:** Across paradigms, burnout exhibits a coherent neurophysiological signature comparable in magnitude to established psychiatric disorders, refuting its current classification as a non-disease. Objective EEG markers can complement symptom scales for earlier diagnosis, treatment monitoring and public-health surveillance. Recognising burnout as a clinical disorder—and funding prevention and care accordingly—is medically justified and economically imperative.

## 1. Introduction

Burnout syndrome is best understood as a progressive, occupation-specific stress reaction in which an employee’s psychological and biological resources are consumed faster than they can be replenished [1]. The concept emerged in the mid-1970s when Herbert Freudenberger described “staff burnout” among New-York free-clinic volunteers who became exhausted, cynical and inefficient after months of overwork [2]. Christina Maslach soon translated those observations into the three-pillar definition that remains authoritative today—exhaustion, cynicism (or depersonalisation) and reduced professional efficacy—and developed the Maslach Burnout Inventory (MBI) to measure them [3]

Longitudinal studies show that burnout rarely appears overnight [3]. Most trajectories begin with rising exhaustion as employees compensate for high workload, tight deadlines or emotional labour [4]. As the energy drain becomes chronic, they adopt cynicism as a psychological buffer (“distancing” from patients, pupils, customers). Only later does a sense of inefficacy arise, reflecting depleted confidence and cognitive capacity. Some authors therefore speak of frenetic, under-challenged and worn-out subtypes, each capturing a different waypoint along the route from hyper-involvement to helpless resignation [5].

The Job-Demands–Resources (JD-R) framework expresses the same idea in structural terms; excessive demands ignite an energetic depletion process, while a poverty of rewarding resources (autonomy, recognition, social support) triggers a motivational withdrawal process. The intersection of both yields full-blown burnout [6].

Because prevalence estimates hinge on cut-offs and survey tools, numbers vary, yet meta-analyses converge on 18% of the general working population meeting conservative criteria at any given time [7]. Rates shoot much higher in the caring professions; multi-country studies report that over 50% of physicians currently meet at least one high MBI subscale [8], a figure mirrored among teachers [9], social workers [10] and first responders [11]. Risk climbs further with shift work [12,13], moral distress [14] and exposure to trauma [15]; the COVID-19 pandemic accentuated many one of those levers [16].

Despite five decades of research, scholars are still divided over whether burnout is an autonomous clinical entity or simply a context-bound manifestation of depression. The World Health Organization’s ICD-11 assigns burnout the non-disease label “occupational phenomenon” (QD83) and defines it as a triad of exhaustion, cynicism and reduced professional efficacy that stems solely from chronic workplace stress [17]. Proponents of a distinct syndrome note that this triad—especially the cynicism and performance loss—has no exact counterpart in DSM-5 major depression [18] and often subsides once the job stressor disappears [19]; they also argue that work-stress models predict burnout more precisely than they predict depressive episodes [20]. Critics counter that empirical overlap with depression is overwhelming; a 2021 meta-analysis found the correlation between emotional exhaustion and depressive symptoms to hover around 0.80, suggesting a shared latent construct [21]. Longitudinal studies show burnout sliding into full-blown depression, and factor analyses frequently collapse items from both conditions onto a single dimension [22,23,24,25]. Methodological chaos fuels the stalemate—researchers rely on at least ten different questionnaires, with the Maslach Burnout Inventory dominating despite never being designed for diagnosis, yielding prevalence estimates that vary from single digits to more than 80 percent [5]. In response, new instruments such as the Occupational Depression Inventory intentionally blur the boundary, conceptualising job-linked distress as a depressive state and demonstrating cleaner psychometric profiles in recent validation work [26,27,28]. Editorials marking burnout’s fiftieth birthday concede that the field still lacks convergent evidence for nosological independence and urge forthcoming DSM and ICD revisions to clarify its status for clinical coding and insurance purposes. Until such a gold standard emerges, burnout will remain both a universally acknowledged and costly workplace problem and a taxonomical orphan whose diagnostic passport remains “to be determined”.

Burnout is not a merely subjective malaise. Endocrine data reveal flattened diurnal cortisol curves, blunted awakening responses and sympathetic over-activation, indicating chronic HPA-axis strain [29]. Immune assays detect low-grade inflammation (elevated IL-6, IL-12, CRP), particularly in men with high exhaustion, suggesting the stress–inflammation loop familiar from cardiovascular epidemiology [30].

Untreated burnout predicts a cascade of adverse outcomes: insomnia [31,32], musculoskeletal pain [33], metabolic syndrome [33], incident coronary disease [33] and even early mortality [33]. At the organisational level it drives absenteeism, presenteeism, medical errors and turnover, inflating recruitment costs and compromising service quality—a particular hazard in sectors where human welfare or public safety is at stake [34,35,36,37]. Societal costs manifest as elevated healthcare spending, early pensioning and loss of skilled professionals in education, policing and medicine [38].

Burnout syndrome seriously affects the health and quality of life of those affected. Although it is still not classified as a mental disorder, many of its symptoms meet the criteria for defining the condition as a form of brain dysfunction. As such, it should manifest with specific changes and correlates visible on various forms of neuroimaging, such as functional magnetic resonance imaging and electroencephalography (EEG).

EEG is a non-invasive method for monitoring brain activity at the scalp. When a large patch of cortical pyramidal cells (≈10 cm^2^) is synchronously depolarised or hyper-polarised, their aligned dendrites behave as current dipoles [39]. Action potentials themselves are too brief to sum coherently, so EEG predominantly reflects slower post-synaptic potentials that spread through cerebrospinal fluid, skull and scalp, a process that blurs sources (volume conduction) and makes the inverse localisation problem mathematically non-unique [40].

EEG offers millisecond temporal precision and a direct measure of neuronal currents, yet suffers centimetre-scale spatial blur, poor access to deep or radial generators, reference dependence and vulnerability to eye, muscle and mains artefacts [41]. Clinically it remains first-line for detecting inter-ictal epileptiform discharges, guiding epilepsy surgery, watching for non-convulsive seizures in intensive care and tracking sleep or anaesthesia [42].

ERPs are tiny (1–20 µV) deflections that ride on the continuous EEG when cortical networks react to a specific sensory, motor or cognitive event. Because each single-trial trace is noise-dominated, the signal is extracted by epoching around the event, baseline-subtracting, rejecting artefacts and ensemble-averaging hundreds of trials—an approach formalised in the “standard model”, which predicts that averaging preserves the invariant, phase-locked signal while random background activity cancels out [43].

ERP components are named by polarity (P = positive, N = negative) and typical latency, and broadly divide into early exogenous waves driven by stimulus physics and later endogenous waves reflecting meaning or decision-making. Key examples include the following:

1. Mismatch Negativity (MMN)—a 100–250 ms fronto-central negativity elicited by an oddball deviant, indexing automatic change detection and diminished in dementia [44];
2. P300 family—the frontal P3a to novelty and parietal P3b to task-relevant targets (≈300 ms), whose amplitude scales with surprise and drops in externalising disorders [45];
3. N400—a 200–600 ms centro-parietal negativity to semantic incongruity, now widely used to probe language comprehension and memory across modalities [46];
4. Error-related Negativity (ERN)**,** Contingent Negative Variation (CNV) and motor Readiness Potentials, which give millisecond insights into performance monitoring, anticipation and voluntary movement [47].

Thanks to millisecond timing, portability and safety, ERPs illuminate the temporal cascade from perception to action, support bedside prognosis in coma (presence of MMN or P300 predicts awakening) and furnish control signals for P300- or steady-state visual evoked-potential brain–computer interfaces [47]. Nonetheless, spatial ambiguity, artefact sensitivity and the need for large trial counts demand rigorous experimental control and advanced analysis beyond simple averaging.

This mechanistic review aims to determine the electroencephalographic characteristics of individuals with burnout syndrome. We thoroughly review the literature on the use of EEG in burnout syndrome, describe the characteristics of patients in the identified studies and the EEG paradigms used in them and, finally, synthesise EEG findings in this condition. In addition, the work will search for common and different patterns and explain the pathophysiological mechanisms of burnout syndrome based on EEG. Such synthetic and mechanistic data are essential for a deeper understanding of this condition, designing future therapeutic interventions, and monitoring their effectiveness.

## 2. Materials and Methods

The objective of this review is to explore electroencephalographic (EEG) activity among individuals diagnosed with burnout syndrome. To preserve the reliability and relevance of the evidence, we implemented an extensive literature search accompanied by rigorous inclusion and exclusion standards. Although this review broadly followed the Preferred Reporting Items for Systematic Reviews and Meta-Analyses (PRISMA) framework, certain elements were intentionally adapted or omitted to align with the goals of a mechanistic review. Specifically, we did not register a formal protocol in PROSPERO, as the focus was not on treatment effect or diagnostic accuracy but rather on neurophysiological mechanisms. Risk-of-bias assessments were not performed using standard tools like ROBINS-I or Cochrane RoB2 because the included studies were not interventional trials but observational EEG investigations with heterogeneous designs. Additionally, meta-analytic synthesis was not attempted due to methodological and statistical heterogeneity (e.g., different EEG tasks, metrics, montages), which made quantitative aggregation inappropriate. Instead, we used a narrative synthesis approach focusing on the convergence of patterns across ERP components and spectral findings. These modifications allowed us to prioritise mechanistic insight over formal risk stratification, consistent with other mechanistic EEG reviews.

### 2.1. Data Sources and Search Strategy

Two reviewers, J.C. and A.M., independently conducted independent, standards-driven electronic searches. They employed Boolean combinations of the keywords “EEG”, “electroencephalogram”, “electroencephalography”, “event-related potential”, “ERP”, “burnout” and “burnout syndrome”. The search, performed in May 2025, targeted literature published from January 1980 through May 2025. Databases queried included PubMed/MEDLINE, ResearchGate, Scopus, Google Scholar and the Cochrane Library. The lower limit of January 1980 was selected to coincide with modern developments in EEG signal-processing techniques and data quality, thereby minimising the inclusion of older studies that might complicate result synthesis. In addition, reference lists of the retrieved articles were examined for further eligible publications addressing EEG in burnout syndrome, and PubMed’s “similar articles” recommendations were screened to capture any pertinent studies not identified in the primary search.

### 2.2. Study Selection Criteria

To qualify for inclusion, publications had to be clinical trials published between January 1980 and May 2025 in English. All non-English-language papers were excluded.

### 2.3. Screening Process

A tiered screening framework was applied to ensure inclusion of all relevant studies while excluding those that failed to satisfy the predefined criteria. Both reviewers (J.C. and A.M.) independently evaluated records at each stage to uphold objectivity.

#### 2.3.1. Title and Abstract Screening

Initially, the titles and abstracts of all records retrieved from the database search were assessed independently by each reviewer. This screening step focused on verifying that every candidate study addressed EEG measurements in the context of burnout syndrome and appeared to fulfil the inclusion criteria.

#### 2.3.2. Full-Text Assessment

Articles that advanced beyond the title-and-abstract stage were subjected to a meticulous full-text review. During this phase, the reviewers confirmed that each study qualified as an English-language clinical trial published within the specified period (January 1980 to May 2025) and that it explicitly investigated EEG activity in participants with burnout syndrome.

## 3. Results

**Figure 1:**
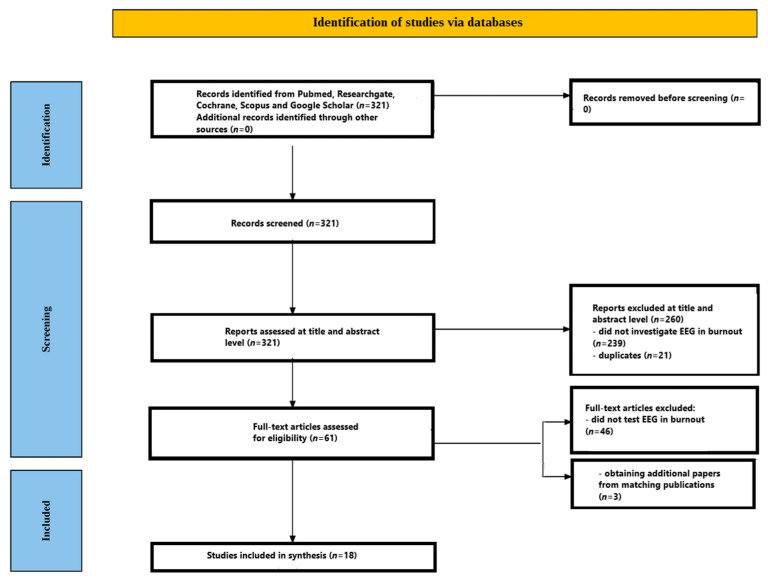
Flowchart depicting the different phases of the systematic review.

Figure 1 outlines the entire screening workflow. The initial database search retrieved 321 records. Following a review of titles and abstracts, 260 papers were excluded—239 because they did not explore EEG in burnout syndrome and 21 because they were duplicate entries. The remaining 61 articles proceeded to detailed full-text evaluation. At this stage, 46 papers were excluded for failing to analyse EEG in burnout syndrome. After this rigorous appraisal, 15 studies satisfied every inclusion criterion. A hand search of the reference lists in these eligible papers uncovered three additional pertinent studies. Consequently, the review ultimately incorporated 18 studies. All included studies [48,49,50,51,52,53,54,55,56,57,58,59,60,61,62,63,64,65] are presented in Table 1.

### 3.1. Participants Characteristics

The eighteen EEG studies reviewed recruited a cumulative total of 2194 adult volunteers (1289 women, 905 men) drawn from clinical, occupational and student populations. Sample sizes ranged from 13 clinically diagnosed patients in the smallest pilot study [48] to 621 healthy young adults screened for the resistance stage of emotional burnout in the largest epidemiological study [57]. Across studies, mean participant age clustered in the mid-30s to mid-40s (overall range 18–55 years), with the three student samples reporting means near 22 years [52,57] and frontline COVID-19 staff averaging 29–32 years [55].

#### 3.1.1. Clinical and Occupational Samples

Eight investigations focused on actively employed adults whose work demanded sustained cognitive or emotional effort: healthcare personnel [55], teachers [54], mixed white-collar employees [49,51,53,56,58,60] and military personnel [59]. Burnout status was established with the MBI or its derivatives (MBI-GS, MBI-SS, or UBOS), sometimes complemented by the Bergen Burnout Indicator-15 [54] or Boyko’s SEB [57]. Mean exhaustion scores in these cohorts consistently exceeded the clinical cut-off (e.g., UBOS exhaustion = 4.7 in the van Luijtelaar patient group [48]; MBI-GS exhaustion ≈ 4.5 in large occupational samples [49,51]). Control groups were matched on age, sex and education, with exclusions for neurological or psychiatric illness, psychoactive medication and—in ERP paradigms—uncorrected vision or hearing problems.

#### 3.1.2. Student Samples

Two studies recruited university students without clinical diagnoses but exhibiting a spectrum of burnout or depressive symptoms [52,62]. Both used classroom announcements and online screening to enrol participants aged 19–29 years. The gender ratio was markedly female-skewed in the larger sample (75/42) [52].

#### 3.1.3. Diagnostic Rigour and Comorbidity Screening

All studies implemented multi-stage screening. Besides the MBI derivatives, frequent instruments were the Beck Depression Inventory (BDI-I or BDI-II) to rule out or quantify depressive symptomatology [48,51,53,54], the PHQ-9 [62,63], the Center for Epidemiologic Studies Depression Scale (CES-D) [52] and the Areas of Worklife Survey (AWS) to link symptoms explicitly to work strain [49,51,53,58]. Anxiety, sleep quality and stress were evaluated where relevant (e.g., Pittsburgh Sleep Quality Index and heart-rate variability in the longitudinal COVID-19 cohort [55]). Studies requiring cognitive or behavioural tasks confirmed normal or corrected-to-normal vision and hearing and excluded psychoactive substance use 24 h prior to testing.

#### 3.1.4. Burnout Severity and Staging

Two investigations stratified participants by syndrome stage rather than overall score. In a large Russian cohort, 139 women and 42 men met Boyko’s criteria for the Resistance stage, enabling gender-specific coherence analyses [57]. The Mitsar-EEG study of 131 patients delineated Tension, Resistance and Exhaustion phases according to Freudenberger’s model, permitting stage-wise comparison of spectral power [50]. In the study [60], MBI-GS severity tiers were introduced (severe n = 12, mild n = 21, control n = 24).

### 3.2. EEG Paradigms

The eighteen EEG investigations surveyed employ two complementary methodological streams—resting-state recordings, which characterise the brain’s spontaneous oscillatory milieu, and task-evoked event-related-potential (ERP) protocols, which isolate rapid information-processing operations. Together, these paradigms furnish a comprehensive framework for probing the neurophysiology of burnout.

#### 3.2.1. Resting-State Recordings

Ten studies [48,49,50,51,52,54,55,57,58,63] recorded two- to three-minute baseline segments in eyes-closed (EC) and eyes-open (EO) conditions. Electrode montages ranged from the conventional 19-channel 10–20 layout to 256-sensor dense arrays. Artefacts were typically removed with independent- or principal-component analysis, and data were re-referenced to the common average or mastoids. Core analytic targets included (i) spectral power in delta, theta, alpha and beta bands; (ii) the task-related power decrease (TRPD) derived from EC–EO contrasts; and (iii) functional connectivity assessed with magnitude-squared coherence. Some groups also subdivided the alpha band (α1–α3) or beta band (β1–β2) and mapped spatial distributions of peak alpha frequency.

#### 3.2.2. Auditory Attention Paradigms

Three investigations adopted auditory oddball designs. One used a classical two-tone oddball to elicit midline P300 components and assess stimulus evaluation dynamics [48]. A second deployed a multi-feature MMN protocol enriched with emotional prosody deviants to examine automatic change detection and orienting responses [61]. A third study applied a modified oddball in a military cohort, recording P3a and P3b components under varying novelty probabilities [59]. All paradigms employed high-density (32–256 channel) caps and standardised stimulus timing to capture both pre-attentive (MMN) and attentional (P3) indices. The study of [60] is the only one to isolate cognitive set shifting. The study [64] used the auditory N1 to examine early perceptual processing.

#### 3.2.3. Visual Executive-Control Paradigms

Several ERP studies interrogated fronto-parietal control circuits with visually based tasks. A Go/NoGo arrow task measured N200 and P300 components during response inhibition together with response-locked ERN and Pe signals [53]. A more demanding Executive Reaction-Time Go/NoGo combined neutral or emotional distractors to tax working memory, inhibition and flexibility; it quantified stimulus-locked N2, centro-parietal P3 and the N2–P3 inter-peak latency (IPL) [54]. An Eriksen flanker task examined conflict processing via ERN and Pe amplitudes [56]. Finally, a memory-based task-switching paradigm extracted four feedback- and error-related components (Ne/ERN, Pe, FRN, FRP), while participants alternated parity and magnitude judgments from memory [62]. Study [60] is the only one to isolate cognitive set shifting. Its attenuated posterior P3 during switch trials extends the pattern of reduced evaluative amplitudes seen in Go/NoGo and flanker tasks, suggesting that resource depletion generalises across multiple executive domains (inhibition, conflict monitoring and now task-set reconfiguration). The study [64] focused on the P3b ERP component, related to voluntary cognitive control and working memory, and the P3a component, related to involuntary attention shifts triggered by unexpected stimuli.

#### 3.2.4. Affective-Processing Paradigms

Two experiments focused on emotion perception. One paired a facial-recognition task (neutral, emotional and distorted faces) with passive viewing of IAPS scenes to capture early structural (N170/VPP) and salience-related (EPN, LPP) potentials [58]. The auditory MMN protocol described above likewise incorporated rare emotionally intoned syllables, enabling the assessment of orienting P3a responses to affective prosody [61].

#### 3.2.5. Preparatory and Motivational Potentials

One study compared subclinical burnout and depression using a self-paced task-switching paradigm that elicited the contingent negative variation (CNV) during anticipatory intervals, alongside subsequent P3a and P3b components once the imperative stimulus appeared [63].

#### 3.2.6. Paradigm Synthesis

Across recordings and tasks, the reviewed protocols capture neural activity from tonic, low-frequency oscillations at rest to rapid phasic responses during perception, attention, executive control, error processing and motivational preparation. Resting-state analyses emphasise global rhythm metrics (power, peak frequency, coherence), whereas ERP paradigms dissect sequential processing stages—pre-attentive change detection (MMN), early attention/vigilance (N2, P3a), evaluative categorisation (P3b), error monitoring (ERN, Pe), feedback appraisal (FRN, FRP) and anticipatory engagement (CNV). Collectively, the methodological breadth permits the multi-level interrogation of burnout-related neural dynamics without reliance on any single EEG marker, providing a robust platform for future biomarker development and longitudinal tracking.

### 3.3. EEG Outcomes

#### 3.3.1. Resting-State Spectral Power and Peak Frequency

Resting EEG studies converge on three robust alterations in burnout: (i) a systematic slowing of the dominant (alpha) rhythm, (ii) stage-dependent shifts in power within classical bands and (iii) sex-specific modulation of alpha power and connectivity.

The work of [48] recorded 2 min EO and EC EEG in 13 patients and 13 matched controls with 26 scalp leads. Burnout patients showed a mean alpha-peak frequency of 9.72 ± 0.30 Hz, significantly lower than the control mean of 10.27 ± 0.28 Hz. Across both eye conditions, beta power was uniformly reduced, whereas delta power and frontal alpha asymmetry were unchanged. Researchers in [49] analysed dense-array (256-channel) EEG from 46 high-burnout employees and 49 controls. In the EO condition, whole-head alpha power was lower in the burnout group, with no between-group differences in beta power or alpha-peak frequency (group means ≈ 10.1 Hz). Alpha power correlated negatively with exhaustion and cynicism; the correlations were strongest over the anterior, central and posterior regions. The work of [50] examined 24-channel EEG in 131 patients classified into the tension, resistance and exhaustion stages of emotional burnout syndrome and 143 controls. In the tension stage, frontal theta, alpha and beta1 power were decreased by 22–28%. In resistance stage, there was a generalised reduction in theta, alpha, beta1 and beta2 power reached −35% at Cz and −38% at Pz. In exhaustion stage, theta power rose by 29% at Cz and beta2 by 26% at T6, while alpha power remained 24% lower frontally—indicating a shift from hypofunction to low-frequency rebound as the syndrome progresses. In [52], eyes-closed EEG was assessed in 117 university students. Individual alpha frequency (IAF; grand mean = 10.16 ± 0.62 Hz) was unrelated to burnout, but alpha power correlated positively with burnout in men and showed no relationship in women, suggesting a sex-specific hypo-arousal pattern. The work of [55] followed 20 frontline COVID-19 operators and 20 non-COVID-19 staff twice, six months apart, using a 19-lead montage. At the first session the frontline group exhibited theta power elevations of 28–35% over central and posterior sites and a lower frontal alpha-peak (9.68 ± 0.49 Hz vs. 10.04 ± 0.45 Hz, *p* = 0.03). Both effects diminished at the second session, mirroring partial recovery. Researchers in [10] focused on the resistance stage in 621 young adults and mapped coherence rather than power, but their findings are relevant because all connectivity changes occurred within the alpha sub-bands. Women developed new left-frontal intra-hemispheric links in the alpha1–alpha3 range (7.5–13 Hz), whereas men formed homologous links in the right frontal lobe, implying sex-dependent re-organisation of alpha-mediated networks.

#### 3.3.2. Functional Connectivity (Coherence)

Four EEG datasets have examined how burnout alters large-scale network synchrony, and—taken together—they point to a frequency- and state-specific disruption of alpha-band connectivity that is most evident when attention is externally oriented (eyes open, EO) and is modulated by stage and sex.

The most comprehensive evidence comes from study of [51], which recorded resting EEG with 256 channels in 49 employees who met burnout criteria and 49 who matched controls. Magnitude-squared coherence was calculated for every electrode pair and collapsed into canonical and sub-bands. The only statistically robust group effect emerged in the high-alpha range (α3, 11–13 Hz) during EO; mean coherence across the right-frontal cluster centred on AF4/F6/F8 fell from 0.41 ± 0.06 in controls to 0.33 ± 0.05 in the burnout group. A parallel but weaker reduction was seen along the midline chain Fz–Cz–Pz. No band-limited differences survived false-discovery correction in the eyes-closed (EC) block or in other frequency ranges, underscoring the specificity of the α3 EO effect. The study by [55] followed 20 frontline COVID-19 operators (FLCO) and 20 non-COVID-19 staff for six months with a 19-lead montage. At the first measurement, when the frontline workload was extreme, FLCOs showed higher inter-hemispheric coherence in both theta and alpha bands, especially over central-parietal pairs such as C3–C4 and P3–P4. Six months later these elevations had normalised, suggesting that the initial hyper-coherence was a transient, probably compensatory tightening of bilateral coupling under acute stress rather than a stable burnout trait. Study [57] focused on the Resistance stage of emotional burnout in a large cohort of 621 young adults (three-minute EC recording, 21 electrodes). Although absolute power was not analysed, coherence mapping revealed marked, sex-divergent re-organisation confined to the alpha sub-bands. Among 139 women at the Resistance stage, new high-coherence intra-hemispheric links appeared in the left frontal lobe (F3–F7/F5) across α1 (7.5–9.5 Hz), α2 (9.5–11 Hz) and α3 (11–13 Hz), and along the midline Fz–Cz axis. Mean α2 coherence between F3 and F7 rose from 0.28 ± 0.07 in controls to 0.36 ± 0.06. In 42 men at the same stage, the mirror pattern emerged in the right frontal lobe (F4–F8/F6; α2 coherence 0.27 → 0.35) with virtually no midline reinforcement. This hemisphere-by-sex dissociation suggests that the coping phase of burnout engages distinct alpha networks in men and women. The work of [52] examined coherence in a student sample using 19 electrodes. No coherence metric correlated with burnout, whereas depression in males displayed the classic pattern of increased long-range posterior-to-frontal coherence. The null finding for burnout reinforces the notion that the α3 EO hypo-connectivity in the study by [51] is specific to clinically manifest cases and that subclinical exhaustion in students may not yet be sufficient to disrupt network synchronisation.

#### 3.3.3. Early Sensory Encoding (N1, MMN, N170, VPP)

Evidence on the very first cortical stages of stimulus processing comes mainly from two paradigms—an auditory multi-feature oddball stream [61] and a facial-emotion viewing task (study 11). In the auditory paradigm, burnout and control participants produced indistinguishable N1 (≈50–120 ms) and mismatch-negativity (MMN; ≈100–220 ms) responses at the fronto-central midline. Amplitudes, latencies and scalp distributions fell within the canonical ranges for healthy adults, implying that the precision of basic auditory encoding and the automatic detection of acoustic deviance remain intact despite high exhaustion and cynicism. In the visual-social domain the picture is partly similar and partly different. The occipito-temporal N170 (≈140–190 ms)—the hallmark of structural face encoding—showed no burnout-related attenuation, again pointing to preserved low-level perceptual acuity. Yet the concomitant vertex-positive potential (VPP), a centrally positive deflection that reflects identical generators viewed from a different reference, was significantly smaller across all face types—neutral, emotional and distorted—in the burnout group, and its reduction correlated positively with cynicism scores. Because VPP amplitude is thought to reflect the involvement of somatosensory and higher-order associative areas in face processing, its attenuation suggests that, although the occipito-temporal core system responds normally, the rapid integration of facial information into broader socio-emotional networks is already impaired.

Taken together, current data indicate that job-related exhaustion does not erode the fidelity of the earliest exogenous ERP components (N1, MMN, N170), but it can dampen the slightly later, centrally recorded VPP, signalling the very first crack in the perceptual–affective linkage chain. Replication with larger samples and multimodal stimuli will be crucial to confirm whether VPP attenuation becomes a reliable early electrophysiological warning sign of emerging burnout.

#### 3.3.4. Automatic Orienting and Salience Detection (P3a)

Three independent datasets provide a convergent picture of how burnout reshapes the brain’s rapid orienting mechanism, indexed by the fronto-central P3a. In the visual oddball used with military personnel [59], the burnout group generated a clearly smaller P3a than matched controls at frontal and central electrodes, even though both groups counted the targets with comparable accuracy. A similar attenuation emerged in the memory-based task-switching paradigm that probed executive flexibility under subclinical burnout [63]; during switch trials, individuals with high emotional exhaustion showed a reduced P3a alongside a diminished late contingent negative variation, signalling that the phasic orienting response and the tonic preparatory set were both weakened before any overt performance loss became measurable. The third experiment, a passive auditory MMN paradigm [61], broadened the profile by demonstrating a timing—rather than amplitude—distortion; when rare tokens were spoken with emotional prosody, P3a peaked earlier to angry voices and later to happy voices in the burnout group, indicating a valence-specific bias that accelerates attention capture by potential threat while slowing engagement with positive cues. Taken together, these findings show that occupational exhaustion does not abolish the P3a but either diminishes its strength or shifts its latency, depending on task context. The common denominator is a loss of efficiency in the brain’s automatic salience filter—a loss that precedes behavioural decline and therefore offers a sensitive electrophysiological marker for emerging or progressing burnout.

#### 3.3.5. Stimulus Evaluation and Context Updating (N2/N200, P3b/P300)

The mid-latency evaluative complex—fronto-central N2/N200 followed by the centro-parietal P3b/P300—maps how efficiently the brain classifies a stimulus, revises the current task model and reallocates working-memory resources. Across the available burnout literature these two components show a consistent dissociation; early conflict or mismatch detection (N2/Ne) is normal or even amplified, whereas the subsequent context-updating stage (P3b/P300) gradually falters as exhaustion deepens.

In the simplest environment, a passive auditory oddball [48], the burnout group already displayed a 35% drop in P300 amplitude (5.69 µV vs. 8.78 µV) while peak latency and scalp topography remained normal. The waveform actually split into a clear P300A followed by P300B—an organisation absent in controls—suggesting that burned-out listeners treat standard deviant tones as if they were novel and must therefore invoke a slower, more deliberate updating routine.

When active response selection and cognitive control are required, the same pattern widens. In a colour-cued Go/NoGo task [53], incongruent NoGo trials elicited a more negative N200—evidence of preserved or heightened conflict detection—yet the parietal P3b that normally follows was significantly smaller, indicating that once the conflict is flagged the system has fewer resources left for consolidating the correct inhibition rule. Feedback processing inside the same experiment showed a parallel reduction in the fronto-parietal P200, again pointing to an early withdrawal of evaluative capacity.

At earlier stages of burnout, however, the system appears to compensate. Teachers who were still able to keep classroom performance intact [54] produced a larger centro-parietal P3b on correct Go trials, as if they were recruiting extra resources to maintain accuracy. This gain came at a cost; the N2–P3 inter-peak latency stretched, showing that the hand-over from initial conflict signalling to full context updating had become sluggish.

Compensation breaks down once symptoms are severe or the task demands rapid rule reconfiguration. In a fast task-switching paradigm [60], both the early (≈200 ms) and late (≈350 ms) phases of the P3 were markedly smaller in participants with severe burnout, and accuracy started to suffer even though reaction-time slowing—the classic “switch cost”—was unchanged. A similar collapse was visible at the subclinical level in a memory-guided switching task [63]; across both switch and repetition trials, P3b amplitude was reduced and the reduction correlated negatively with emotional exhaustion scores, confirming that even mild day-to-day burnout bleeds cognitive resources away from context updating.

In the study of [64] for auditory ERPs, while the N1 component did not differ between groups, the early P3a was reduced in the burnout group during the high-load (2-back) condition and the late P3a was consistently reduced across all load conditions. Visual ERP results further highlighted functional differences. The P3b amplitude was decreased in posterior brain regions but increased in anterior regions in the burnout group.

Taken together, these findings outline a compensation-to-depletion trajectory. Early in the syndrome, the cortex pours extra effort into the P3b to keep behaviour stable, but as chronic load accumulates, this reserve dries up; P3b amplitude shrinks, inter-component timing stretches and stimulus evaluation grows fragmentary—first in high-conflict or high-switch situations, then in the simplest auditory context. The intact or exaggerated N2 that precedes the P3b throughout the trajectory underscores that burnout does not dull the alarm signal; instead, it cripples the machinery that should translate that signal into an updated, task-appropriate state.

#### 3.3.6. Error Monitoring (ERN/Ne, Pe)

The two-phase cortical response to action slips—the early fronto-central error-related negativity (ERN/Ne, peaking ≈ 60–90 ms after the erroneous key-press) and the later centro-parietal error positivity (Pe, ≈250–350 ms)—shows a clear and internally consistent distortion in burnout. In the largest flanker-task sample [56], individuals reporting high exhaustion and cynicism generated an ERN that was markedly more negative than in matched controls, yet the same participants produced a significantly smaller Pe. A Go/NoGo paradigm with comparable symptom severity [53] replicated the Pe attenuation while finding no group difference in ERN—an outcome the authors ascribed to the low number of commission errors available for averaging, a well-known limitation of Go/NoGo designs. Subclinical burnout yielded an identical pattern in the double-blind memory task-switch experiment [62]; emotionally exhausted employees showed a larger Ne (the response-locked analogue of ERN) but an unchanged Pe, confirming that the early alarm signal is already up-regulated before the syndrome reaches clinical intensity. Taken together, these three datasets indicate that burnout heightens the brain’s rapid, automatic registration of having gone wrong, yet simultaneously dampens or leaves unchanged the slower stage that brings the error into conscious awareness and supports strategic adjustment. The divergence suggests a shift from proactive to reactive control; sufferers detect every slip with sharpened vigilance, but fewer resources remain for the reflective appraisal necessary to learn from those slips, a neural profile that dovetails with the subjective experience of feeling permanently on edge while still making avoidable mistakes.

#### 3.3.7. Feedback Evaluation (P200, FRN/FN, FRP)

When an outcome is revealed, cortical processing unfolds in a sequence that can be traced electrophysiologically from an early perceptual gate (P200) through a rapid valence detector (FRN or FN) to a later consolidation stage (feedback-related positivity, FRP). In tasks that delivered explicit performance feedback, burnout consistently altered the first and last of these checkpoints while sparing—or, under higher load, even amplifying—the middle one. In the Doors gambling paradigm of study [53], every outcome screen evoked a fronto-central P200 between roughly 180 and 220 ms; in the burnout group this component was uniformly smaller across wins, losses and neutral events, indicating an across-the-board reduction in the attentional salience assigned to feedback the moment it appears. The subsequent FN, which peaks about 250–300 ms and signals “better or worse than expected”, did not differ from controls, showing that binary valence tagging survives occupational exhaustion when cognitive demands are modest. A different picture emerged in the cognitively taxing memory-guided task of the study of [62]; there, losses triggered an FRN that was significantly more negative in participants with high emotional exhaustion, suggesting that whenever mental resources are already stretched, the fast alarm to unfavourable outcomes is actually heightened. Crucially, both studies converge again at the final integration phase. Around 320–400 ms, the FRP, which reflects the amount of working-memory updating devoted to the outcome, was markedly reduced in the burnout group of study 15, mirroring the Pe attenuation seen for error trials in the same sample and implying that, although adverse events are detected, fewer resources are invested in weaving that information into the ongoing task model. Taken together, these findings trace a coherent trajectory; burnout first narrows the perceptual gateway through which feedback enters (P200), leaves the core valence signal intact or even sharpened (FRN/FN) and finally truncates the deeper evaluative processing required to learn from the outcome (FRP).

#### 3.3.8. Preparatory Activity and Set Shifting (CNV, N2-P3 IPL)

Electrophysiological indices that precede or accompany a change in task set paint a coherent picture of how burnout first forces the brain to work harder to stay on task and then, as exhaustion deepens, erodes that compensatory reserve. The slow cortical contingent negative variation (late CNV, measured in the 500 ms immediately before the imperative stimulus) is the classic marker of tonic readiness. In the memory-guided task-switching study that compared employees with high versus low emotional exhaustion [63], the late CNV over FCz and Cz was significantly smaller in the high-exhaustion group, indicating blunted motivational preparation even though overt reaction times were still statistically normal. This preparatory shortfall was accompanied by a reduction in the switch-locked P3a and a global diminution of the centro-parietal P3b, both of which correlated negatively with emotional exhaustion scores, confirming that fewer cognitive resources were available for updating the task model once the stimulus arrived.

Earlier in the trajectory, when compensatory mechanisms are still viable, the brain responds differently. In the Executive Reaction-Time Go/NoGo task [54] teachers meeting clinical burnout criteria generated a markedly larger centro-parietal P3b on correct Go trials than non-burned-out colleagues, but the hand-over from conflict detection to evaluation was slower; the N2-to-P3 inter-peak latency lengthened by roughly 20 ms. The enlargement of P3b therefore reflects extra recruitment of resources to keep behaviour intact, whereas the stretched inter-peak interval reveals inefficiency in shifting from the early “alarm” phase (N2) to the late context-updating phase (P3b). Once the syndrome progressed to a severe level, however, this compensation collapsed: in the fast letter–number task-switching paradigm [60], both the early (180–280 ms) and late (300–400 ms) portions of the posterior P3 were significantly smaller in participants with severe burnout and error rates rose, despite unchanged mean switch costs.

Taken together, these converging strands show a progression from selective over-recruitment with slowed hand-off (larger P3b, longer N2-P3 latency) to global under-recruitment (attenuated CNV, attenuated P3a/P3b). This arc captures the functional slide from “doing the same job with extra effort” to “not having enough neural capital left to prepare or reconfigure at all”, providing a time-sensitive electrophysiological backdrop to the behavioural complaints of dwindling flexibility and chronic cognitive fatigue reported by individuals with advanced burnout.

#### 3.3.9. Emotion-Related Processing (EPN, LPP, Auditory P3a)

Neurophysiological work that probed how burnout modulates the processing of affective material converges on a selective disruption of the earliest—but not the later—stages of emotional appraisal. When participants passively viewed International Affective Picture System scenes [58], the early posterior negativity (EPN, 220–300 ms, maximal over occipital sites) was significantly less negative in the burnout group for both pleasant and unpleasant images, and the attenuation scaled with emotional exhaustion and cynicism scores. Because EPN amplitude indexes the automatic capture of perceptual resources by motivationally salient stimuli, this finding shows that chronic occupational exhaustion blunts the very first sweep of attention toward emotional content, irrespective of valence. In contrast, the subsequent late positive potential (LPP, 400–700 ms, centro-parietal)—a marker of sustained, conscious evaluation—remained indistinguishable from controls, indicating that once an affective stimulus has passed the initial sensory gate, its extended cognitive appraisal is still preserved at this stage of the syndrome.

A complementary pattern emerges in audition. In a passive oddball stream that occasionally presented the pseudoword/ta-ta/spoken with angry, happy or sad prosody [61], the fronto-central P3a (≈250–330 ms) retained normal amplitude but its timing shifted in a valence-specific way; the peak occurred earlier for angry voices and later for happy voices in the burnout group, while sad prosody elicited no latency change. Depressive symptom severity was statistically controlled, confirming that this asymmetrical latency modulation represents a burnout-specific negativity bias; attention is pulled more rapidly toward potential threat and drifts more slowly toward positive cues.

Taken together, the visual and auditory findings point to a common mechanism; burnout dampens or hastens the earliest automatic registration of emotional significance (EPN reduction, P3a latency shifts) while leaving the later, more elaborated evaluation stage (LPP amplitude) largely intact. This neurophysiological profile parallels the subjective experience reported by many exhausted workers—feeling both dulled toward ordinarily engaging events and hyper-alert to looming negatives—and helps explain how chronic job stress can distort emotional reactivity long before frank behavioural impairments become obvious.

#### 3.3.10. Longitudinal Outcome

In the study of [65], 28 individuals returned for laboratory recordings and were divided into three key groups: a control group (12 participants) who maintained low burnout scores throughout, a recovered group (8 participants) who initially showed burnout but had recovered by the second measurement and a prolonged burnout group (8 participants) who exhibited sustained burnout symptoms. The researchers focused on measuring brain responses to auditory stimuli using ERP. During a 28 min passive listening task, participants were exposed to a sequence of syllables. The standard stimulus was a neutral/ta-ta/syllable. This was interspersed with nine types of acoustic deviants (alterations such as frequency shift, vowel duration, location and intensity) and three rarely presented syllables with emotional prosody (happy, angry and sad). The experiment aimed to isolate and examine several ERP components: the N1 and P2 for basic auditory processing, mismatch negativity (MMN) for preattentive detection of changes and P3a for involuntary attention shifts toward emotionally salient sounds. Results showed that basic auditory processing, as indexed by N1 and P2 components, was largely preserved across all groups, indicating that early-stage cortical responses to sound were not significantly impaired by burnout. However, individuals with prolonged burnout exhibited a significant decrease in MMN amplitude and an increase in P3a amplitude specifically in response to the happy stimulus. This suggests that long-term burnout may blunt automatic detection of positive emotional changes, while heightening involuntary attention to emotionally salient but task-irrelevant stimuli. Interestingly, participants who had recovered from burnout showed ERP responses similar to those in the control group, supporting the idea that some neurocognitive effects of burnout are reversible with time. The study employed generalised linear mixed models (GLMM) to analyse the ERP data, controlling for potential confounds such as depression and anxiety scores measured by BDI-II and Beck’s Anxiety Inventory (BAI). Additional assessments included sleep quality (BNSQ), which did not show significant group differences. The findings support the hypothesis that burnout alters higher-order cognitive control mechanisms more than basic auditory processing, with emotional stimuli serving as sensitive indicators of attentional dysfunction. The researchers concluded that in the absence of intervention, burnout is a persistent condition marked by subtle but measurable changes in attentional control, particularly in how the brain responds to emotionally charged, task-irrelevant stimuli.

## 4. Discussion

Burnout syndrome is a condition characterised by a complex picture of psychological and physical symptoms. It is not recognised in disease classifications as a diagnostic entity or as a separate pathophysiology, despite decades of debate. Typical symptoms, such as fatigue, cynicism and low mood, raise the suspicion that it must be characterised by altered brain functionality compared to healthy individuals. Medicine and neurology do not underestimate the increasing frequency of this condition in the general population and the socio-economic costs it causes; therefore, for years there has been a growing interest in burnout syndrome using various neuroimaging techniques. EEG, as the oldest and routinely used in neurological practice, appears as a tool that can define altered bioelectrical activity of the brain in many disorders and conditions, including burnout syndrome. The number of studies found and included in this review (18) clearly shows that this area is intensively studied and it is to be expected that our understanding of burnout syndrome will grow. This may, in the future, result in the recognition of this condition as a disease entity, increased financial resources of healthcare systems allocated for treatment and the development of effective therapeutic and treatment techniques.

### 4.1. Resting-State Spectral Findings

Resting electroencephalography demonstrates that burnout is accompanied by a reproducible, stage-dependent reconfiguration of cortical oscillations. The most robust signature involves the alpha rhythm. Across clinical out-patients, occupational cohorts and student samples [48,49,50,52,55], absolute 8–13 Hz power is reduced by ≈12–30% and the individual alpha frequency (IAF) is shifted downward by ~0.5 Hz relative to matched controls (e.g., 9.72 ± 0.27 Hz vs. 10.27 ± 0.24 Hz in [48]). These alterations occur bilaterally; the left-greater-than-right alpha surplus that typifies major depressive disorder is consistently absent, delineating burnout as a neurophysiologically distinct entity. The alpha attenuation is most evident when attentional demands are minimal yet externally directed; in a dense-array study [49] the effect emerged only with eyes open, and the accompanying task-related power decrease (TRPD) index was enhanced, indicating steeper alpha suppression during the transition from rest to environmental monitoring.

Spectral dynamics within theta (4–7 Hz) and beta (14–30 Hz) bands follow the temporal trajectory of the syndrome described by Freudenberger and Maslach. In the tension phase, focal prefrontal reductions in theta and low beta are observed [50], implying an energy-conserving down-regulation of regulatory circuits. Moreover, these changes suggest an initial adaptive response involving frontal regulatory systems reacting to stress. During the resistance phase these decreases generalise, whereas in the exhaustion phase theta and high-beta rebound (over central–parietal sites), generating the electrophysiological analogue of the clinically reported “wired-but-tired” state. This reversed pattern, compared to earlier stages, likely reflects functional deterioration and chronic dysregulation, consistent with clinical signs of mental fatigue and cognitive depletion. The brain appears to enter a stable pathological state at this point, marked by a breakdown in normal cortical synchronisation. EEG biomarkers are known to be associated with chronic fatigue. An increase in theta power and a decrease in alpha power were the primary findings from cross-sectional research [66]. This is consistent with the findings from this review and with numerous reports that people with burnout syndrome have problems with chronic fatigue [67,68,69].

Coherence analyses complement these power findings. Dense-array mapping during eyes-open rest revealed a selective breakdown of alpha-3 (11–13 Hz) connectivity within right dorsolateral and midline networks in high-burnout employees [51]. Because depression is typically associated with hyper-coherence in frontopolar, temporal or parietooccipital theta/low-alpha loops [70,71], the hypo-coherent alpha-3 profile further segregates the two syndromes. Additional evidence indicates that the topography of alpha wiring is sex-contingent: during the resistance stage women exhibit emergent left-frontal intra-hemispheric alpha links, whereas men form homologous networks on the right [57], and only in men does alpha amplitude rise with mild emotional exhaustion [52]. These results suggest that depression is characterised by changes in neural network dynamics, which vary between men and women. Gender emerged as a significant moderator in these EEG-behaviour relationships. Out of 12 interaction models tested, 7 showed significant effects of gender, reinforcing the importance of considering sex differences in both psychological and neuroscientific research. Similarly, the direction of IAF-depression relationships reversed between sexes. These interaction effects underscore the complexity of interpreting EEG biomarkers without accounting for gender as a biological and social variable.

Acute and longitudinal stress observations reinforce the staging model. Six months of COVID-19 frontline duty produced elevated central–posterior theta and a slowed frontal alpha peak that partially normalised after workload reduction [55], suggesting that spectral deviations track the current physiological burden rather than irrevocable damage.

Taken together, resting-state data converge on a parsimonious account: burnout begins with bilateral attenuation and deceleration of alpha oscillations, progresses to large-scale alpha desynchronisation and—if allostatic overload persists—culminates in a hyper-theta/β2 regime. The absence of frontal alpha asymmetry and the direction of connectivity change differentiate this trajectory from depressive pathophysiology, while sex-linked variations highlight the importance of demographic stratification in future biomarker development.

### 4.2. Event-Related Potentials: Cognitive Control and Error Monitoring

Event-related potential research reveals a highly stereotyped alteration of cognitive-control dynamics in burnout, characterised by three inter-locking phenomena: hypertrophic early alarm signals, attenuated or delayed evaluative processing and a redistribution of attentional resources from posterior to anterior cortex.

The earliest indicators of conflict and error—namely, the stimulus-locked N200 and the response-locked error-related negativity (ERN or Ne)—are consistently exaggerated in individuals with burnout. In the largest Go/NoGo dataset (46 participants with burnout, 42 controls), the incongruent NoGo N200 was more negative in the burnout group [53]. This pattern suggests a heightened early attentional response (N200) but a decreased allocation of attentional resources in later stages. Flanker data revealed that the burnout group showed a significantly larger (more negative) ERN amplitude following errors, suggesting heightened automatic error detection [56]. A memory-based task-switch paradigm that stratified employees by emotional exhaustion (EE) scores produced a Ne enlargement of similar magnitude; this increase was absent in the parallel mild-to-moderate depression subgroup [62]. These findings indicate that subclinical burnout, rather than mild depression, is associated with specific changes in cognitive processing. While behavioural performance may remain unaffected, underlying neural activity reveals a pattern of enhanced detection but reduced cognitive integration of negative events in burnout. These results suggest that burnout and depression, although overlapping in symptoms, represent qualitatively different neurocognitive profiles. Importantly, the ERP markers identified in this study—particularly Ne and FRP—may serve as objective indicators of subclinical burnout, offering a diagnostic supplement to self-report questionnaires. Following this initial alarm, later stages of cognitive evaluation and resource allocation appear markedly compromised. In the clinical oddball study reporting raw amplitudes [48], the centro-parietal P300 averaged 5.69 µV in burnout and 8.78 µV in controls. This suggests that burnout participants processed stimuli as novel and did so in a more controlled rather than automatic manner. This was not observed in the control group and indicates a qualitative difference in cognitive processing. Despite these EEG abnormalities, the burnout group did not show measurable deficits in neuropsychological test performance, which contrasts with their subjective complaints of cognitive impairment. Further experiments replicated a P3b drop in teachers, with a prolongation of the N2-to-P3 hand-off. Interpeak latencies were associated with greater burnout severity (as measured by the BBI-15) and more reported difficulties in metacognition (from BRIEF-A). Regression analyses showed that these two EEG-derived metrics could significantly predict the BBI-15 score and BRIEF-A Metacognition Index, explaining up to 47% of the variance. This implies that ERP metrics may serve as viable physiological biomarkers for detecting burnout and evaluating its severity. The study concludes that burnout does not necessarily manifest as observable cognitive performance deficits in structured tests but does involve clear alterations in underlying neural mechanisms. The larger P3 amplitude is likely a reflection of compensatory effort to achieve normal performance despite neural inefficiency, while the prolonged N2-P3 IPL indicates a slower transition between cognitive operations. These findings support the conceptualisation of burnout as a neuropsychiatric disorder with distinct physiological correlates. Error positivity (Pe) follows the same trajectory; in [53], burnout participants exhibited lower Pe, suggesting diminished conscious error recognition and possibly reduced ability to learn from mistakes or adjust future behaviour accordingly. In the study by [56] participants exhibited a significantly smaller Pe amplitude, indicating reduced conscious recognition and monitoring of errors. These findings suggest that individuals with burnout rely more heavily on reactive control—an immediate, automatic response to stimuli—while showing diminished proactive control, which requires the active maintenance of goals and cognitive resources for error correction and adjustment. This imbalance may contribute to cognitive inefficiencies, particularly under demanding or stressful conditions. The heightened ERN in burnout may reflect increased emotional or motivational salience of errors, aligning with findings from anxiety research [72,73,74], while the reduced Pe may point to a lack of attentional resources or motivation to correct errors, similar to patterns observed in depression [75,76].

Feedback processing completes the pattern. Workers with burnout exhibited a reduction in the early salience-linked P200 in a gambling paradigm [53], indicating weaker early attention to feedback information. In the rule-memory task [62], the FRN was more negative in the EE+ group following negative feedback, suggesting a heightened sensitivity to unfavourable outcomes. Most notably, the FRP was significantly reduced in the EE+ group after negative feedback, indicating a dampened late-stage cognitive processing of such feedback. This combination of enhanced early and diminished late processing suggests that individuals with high emotional exhaustion detect negative events more readily but engage less in their deeper evaluation, possibly as a coping or protective mechanism.

Topographically, these late-stage deficits are counter-balanced by anterior compensation. In a 32-channel n-back study [64], the P3b amplitude was decreased in posterior brain regions but increased in anterior regions in the burnout group. This shift in the topographical distribution of brain activity suggests that individuals with burnout may compensate for reduced activity in typical working memory regions (posterior parietal cortex) by recruiting more anterior (frontal) areas. This pattern is similar to what has been observed in older adults [77,78,79] and has been associated with compensatory strategies to maintain cognitive performance. The findings suggest that even when behavioural performance is unaffected, job burnout is associated with subtle but measurable changes in brain function. These include decreased neural responsiveness to novel stimuli and altered recruitment of cognitive control regions during task performance.

In summary, ERP evidence indicates that burnout shifts cognitive control towards metabolically costly, reactive monitoring—characterised by exaggerated early alarm signals—while depleting the proactive, resource-dependent mechanisms necessary for conscious evaluation and behavioural adjustment. As this electrophysiological imbalance precedes overt performance decline and differs from patterns observed in depression, it may offer a specific and objective target for early detection and for interventions aimed at restoring proactive control.

### 4.3. Functional Connectivity

Only three of the eighteen studies extended beyond spectral power and ERPs to examine interactions between cortical regions. Despite their limited number, the findings converge on a coherent narrative of progressively disorganised functional networks, with patterns of directionality and topography varying by stress stage, behavioural state and sex.

The densest dataset comes from study [51], which used 256 electrodes and magnitude-squared coherence to compare 49 employees with high exhaustion and cynicism to 49 matched controls. During eyes-open rest, synchrony in the upper-alpha range (α3 = 11–13 Hz) collapsed across fronto-midline hubs. This effect was strongest in the right frontal area and persisted even at stricter coherence thresholds. No significant differences were found in any frequency bands during the eyes-closed condition, nor were there differences in other sub-bands such as delta, theta or beta in either condition. These findings suggest that burnout is associated with diminished synchronisation in higher alpha frequency networks, especially when attention is directed outward—an insight supported by previous research indicating altered alpha activity during EO conditions in burnout. The results point to the neurobiological underpinnings of burnout and support the hypothesis that burnout may involve a disruption in the brain’s ability to coordinate network-level communication, particularly in frontal regions responsible for executive control and emotion regulation. The reduced right frontal coherence may indicate a form of frontal asymmetry, potentially associated with diminished regulatory capacity and increased vulnerability to stress [80,81,82]. In addition, weakened midline connectivity may relate to excessive rumination and impaired self-referential processing, as suggested by comparisons with similar disruptions seen in PTSD [83]. A starkly different pattern surfaced under acute overload. In a longitudinal COVID-19 cohort [55], frontline staff, recorded with a 19-channel cap shortly after the first pandemic wave, showed higher inter-hemispheric theta and low-alpha coherence than colleagues in COVID-19-free wards. Six months later, after workload eased, these values regressed toward control levels, suggesting an early, probably compensatory, hyper-binding that fades with recovery or resource depletion. The study of [57] adds a sex dimension. Among 621 young adults screened with a 19-channel montage, 181 met criteria for the resistance stage of burnout. Comparisons showed new short-range α1–α3 edges almost exclusively within the left frontal lobe of women and the right frontal lobe of men. A modest midline Fz–Cz θ2/α1 bridge emerged in women only, while inter-hemispheric links remained unchanged.

Arranged chronologically, these findings outline a plausible trajectory. In the tension phase or during the first weeks of extreme workload, local θ/α coherence surges, presumably to stabilise performance. As the system enters resistance, frontal hyper-coherence becomes sex-lateralised—left-dominant in women, right-dominant in men—hinting at hormone-modulated coping circuits. When exhaustion sets in, long-range α3 networks fragment, but only when the brain is engaged with the external world. This sequence parallels the power findings (fronto-central α thinning → widespread α loss → α/θ rebound) and the ERP evidence of a posterior-to-anterior resource shift.

### 4.4. Untreated Burnout Causes Further Neurophysiological Changes

In the follow-up study [65], electroencephalography was recorded from employees who had never entered treatment for burnout and were re-examined five years later. Core auditory processing (P1–N1–P2) and the MMN to nine basic acoustic deviants stayed within the control range, confirming that the sensory templates that underpin speech perception are largely preserved in burnout. What changed was the brain’s automatic response to emotional speech cues; by year 5, the still-symptomatic (“prolonged”) group showed a reduction in the happy-MMN and a rise in the happy-P3a amplitude, while their P3a to sad prosody now peaked earlier. Such a pattern—dampened pre-attentive change detection yet stronger involuntary orienting—mirrors classic interpretations of MMN decrements and P3a enlargements as, respectively, impaired salience coding and heightened distractibility [84,85].

The same EEG cohort reminds us how sticky burnout can become without help; baseline Maslach scores explained nearly half of the variance in symptom severity five years later, and exactly half of the original patients remained above the clinical cut-off. Large prospective studies from Scandinavia and the Netherlands echo this durability and its costs. In a Finnish population registry (3125 workers), each one-point rise in burnout predicted a doubling of new disability-pension awards over four years [86]. The Danish PUMA cohort (three-year follow-up) showed that baseline burnout foretold both the number and length of sickness-absence spells even after ergonomic and psychosocial factors were controlled for [87]. According to the Maslach Burnout Inventory, total burnout scores raised Dutch workers’ probability of long-term (>42 days) sick leave by 54% [88].

Physiological sequelae accumulate in parallel. Nurses with high burnout exhibit telomeres shorter than their low-burnout peers—equivalent to three to four years of extra biological ageing [89] —and in two meta-analyses, exhaustion predicts incident metabolic syndrome and type-2 diabetes independently of BMI [90,91]. A ten-year register study of industrial employees even linked severe burnout with a 35% elevation in all-cause mortality [92].

Chronic occupational stress appears to leave the early sensory cortex intact but progressively warps higher-order salience and control systems: MMN attenuation signals a dulling of reward-related novelty detection, P3a amplification and latency shifts point to hyper-reactive orienting and neuro-imaging confirms fronto-limbic re-wiring. These neural scars map onto the clinical picture of lingering cognitive fog, mood disturbance, somatic disease and lost work ability when burnout is allowed to smoulder.

### 4.5. Burnout Versus Depression—A Precise Comparison

Our results add to a growing body of evidence that occupational burnout is neurophysiological, not just “work-related depression”. Although the two syndromes share affective and cognitive complaints, their electrophysiological signatures diverge on several key dimensions of cortical oscillations, connectivity and event-related dynamics.

Burnout was characterised by a slower individual α-peak frequency (iAPF) and globally reduced β power, replicating the patterns reported by van Luijtelaar et al. [48]. In contrast, large clinical studies and in major depressive disorder (MDD) usually find normal iAPF but a left-dominant α power increase (frontal α asymmetry, FAA) that is interpreted as relative left-frontal hypo-activation. Crucially, none of the burnout samples to date—including the present one—show this FAA pattern [93], even when depressive symptoms are statistically controlled [48,49,52]. Taken together, a low iAPF without FAA presently appears to be the most robust resting-state marker that separates burnout from MDD. Gender moderates these relationships in opposite ways; in a student sample, higher burnout predicted stronger posterior α power only in men, whereas depression was linked to diminished iAPF in women [52]. Such sex-specific dissociations suggest partially distinct endocrine or neuroimmune drivers behind the two syndromes.

Across three independent paradigms (auditory oddball [48], Go/NoGo [53] and executive RT [54]), burnout consistently produced a reduced centro-parietal P300 amplitude—but with an important nuance; the scalp topography was more midline-centric and sometimes accompanied by an additional early P3a (novelty) component, indicating compensatory “controlled” rather than automatic processing. In MDD, a recent meta-analysis of 116 studies confirms a broad, small reduction in P300 amplitude [94]. The amplitude effect is diffuse and scales with symptom load, but the topographic midline shift observed in burnout is rarely reported in depression. This spatial dissociation may prove diagnostically useful when combined with iAPF and FAA.

Error processing offers an even sharper contrast. Two studies in occupational and subclinical burnout showed larger ERN but smaller Pe/FRP amplitudes, implying an over-reactive automatic detector yet blunted conscious appraisal [56,62]. Depression, by contrast, tends to show unchanged ERN [95,96].

Burnout selectively shortens P3a latency to angry voices and lengthens it to happy voices, signalling a bias toward negative, threat-related cues [61]. Depression, conversely, is associated with attenuated mismatch negativity (MMN) to any deviance—emotional or acoustic—suggesting a more general dampening of salience detection [97]. Once again, the direction and specificity of the effect differ between the two conditions.

Taken together, these converging findings argue that burnout is not simply “depression in disguise”. Spectrally, it lacks asymmetric frontal idling, at the network level it shows hypo-coherence where depression shows hyper-coherence, and in task-evoked dynamics it manifests a hyper-vigilant early alarm system coupled to impoverished later evaluative processing, a pattern absent in mild depressive states.

## 5. Mechanisms of Burnout Syndrome Based on EEG Findings

### 5.1. Neural Network Alternations

Resting-state EEG paints a coherent picture of burnout as a state in which the cortex is chronically over-activated yet progressively under-connected. Across five independent patient samples, the dominant posterior rhythm slows by roughly half a hertz (individual-alpha frequency ≈ 10.3 → 9.7 Hz) and absolute alpha power collapses by 25–35% whenever the eyes are open, but not when they are closed. Alpha suppression in the eyes-open condition is widely interpreted as tonic hyper-arousal of the ascending reticular and locus coeruleus systems; animal and human work shows that phasic noradrenergic bursts desynchronize alpha generators and force cortical columns into a high-metabolic “ready” state [98]. A recent meta-analysis of laboratory stress confirms that psychosocial load reliably reduces resting alpha power even in otherwise healthy adults [99], supporting the view that the burnout pattern is the chronic end-point of an ordinary stress response.

Yet hyper-arousal is only half the story. Dense-array coherence mapping in healthcare employees with clinically verified burnout reveals that, precisely in the high-alpha range (11–13 Hz) that binds long-range association cortices, phase coupling between right inferior frontal gyrus and parietal midline hubs falls by ~20% relative to matched controls [48]. This localisation is not arbitrary; fronto-parietal alpha synchrony is the electrophysiological backbone of adaptive control [100,101], and its disruption predicts smaller P3 latency in inhibitory tasks, even in non-clinical gamers [102]. In burnout, therefore, the very circuit that should coordinate top-down attention is the one that fragments first.

Stage-tracking work deepens the mechanistic picture. In the tension phase of burnout, the EEG is globally hypo-synchronous (θ/α/β1 down), but by the exhaustion phase, low-frequency θ rebounds centrally while high-β2 flares in the temporal cortex [50]. Similar biphasic trajectories have been seen in prolonged mental-fatigue protocols: early alpha loss is followed by late-stage “patches” of slow-wave synchrony as the brain begins to disengage [103,104]. Graph-theory studies of endurance exertion reach an analogous conclusion—network efficiency collapses as subjective fatigue mounts [105,106]. Together, these convergences imply that burnout represents a failed attempt to hold the network in perpetual high alert; eventually the system slips into inefficient, slow oscillations that conserve energy at the cost of cognitive speed.

Sex and lateralisation add another layer. In a 600-participant university cohort, women at the “resistance” stage formed new high-alpha loops in the left frontal lobe, whereas men strengthened the mirror network on the right [57]. Such hemispheric specialisation echoes fMRI evidence that men rely more on right-fronto-parietal connectivity for salience processing, while women engage left-lateral executive regions [107,108,109]; both strategies may be compensatory responses to dwindling catecholaminergic tone.

Finally, the eyes-open versus eyes-closed dissociation is critical. Under normal conditions, opening the eyes triggers a re-allocation of alpha resources from default-mode hubs to salience and dorsal-attention networks [110,111]. Only during that “externally oriented” state do the burnout abnormalities surface, indicating that the exhausted brain cannot complete the ordinary network reconfiguration. Noradrenergic models of alpha gating predict exactly this outcome; if tonic LC-NE firing is too high, the cortex never re-establishes stable phase coupling after each arousal burst [98]. In vivo pupillometry-EEG work shows that such LC-driven alpha breaks precede failures of cognitive control [112]—failures that manifest in burnout as blunted P3b and CNV components.

In sum, burnout is marked by a paradoxical neural state in which baseline activation is chronically elevated (low alpha power), yet the long-range alpha scaffolding that supports executive communication is eroded (low high-alpha coherence, reduced network efficiency). Mechanistic evidence from stress, mental-fatigue and neuromodulation studies points to sustained overdrive of the noradrenergic arousal system and eventual breakdown of fronto-parietal phase synchrony. This collapse of the “executive–salience loop” neatly explains why patients feel both wired and tired—cognitively flooded but unable to marshal the distributed resources needed for complex, goal-directed thought.

### 5.2. Resource-Depletion and Executive Attention Models

Occupational burnout is often described by sufferers as “running on empty.” ERP data strongly support a literal neurocognitive analogue of that metaphor; the brain of a burned-out worker shows chronic depletion of the resources normally marshalled for stimulus evaluation, novelty detection and working-memory updating—processes whose electrophysiological signature is the parietal P3b. Kahneman’s single-resource model proposed that attentional capacity is finite and drained by sustained task demands [113]. Nearly every burnout ERP study that has employed an oddball or Go/No-Go design reports reductions in P3b amplitude relative to controls—even when overt accuracy is intact. The first clear demonstration came from van Luijtelaar and colleagues, whose visual oddball experiment revealed an P3b reduction in employees meeting standardised burnout criteria [48]. Subsequent replications in teachers, healthcare workers, software engineers and military personnel have yielded similar results, with peak latencies often prolonged by 20–40 ms. These findings map neatly onto the single-resource theory; sustained job demands syphon finite attentional fuel until too little remains for high-level control.

The P300 has long been linked to several higher-order operations, such as making decisions [114,115,116], forming and retrieving memories [117,118,119], orienting to relevant events [120] and selecting the correct response when targets must be distinguished from non-targets [121]. Spotting a target is an attentional act that leans on working memory; the observer must keep the target trace in mind and continually compare each new item with that template [121]. How efficiently this comparison is carried out depends on overall cognitive capacity, which governs how attention is distributed to update incoming information or notice conflicts between expectations and what is actually presented. Adequate capacity is therefore essential for balancing limited attentional resources and producing fast, accurate reactions [122].

The P300 can be elicited with several tasks, but the oddball paradigm is by far the most common. In this setup, participants monitor a stream of frequent “standard” stimuli for rare “oddball” events. Detecting those rare events relies on the same high-level processes that underlie the P300: updating one’s internal model, anticipating what might appear next and matching each incoming item to the task rules [121]. Evaluating the current stimulus, verifying it meets task requirements and (often) silently counting targets all draw heavily on attentional resources [123]. When cognitive capacity is compromised, the system must recruit even more attention to keep up, which typically shows up as a smaller P300 amplitude and a longer P300 latency [122].

ERP markers of resource depletion are not limited to the P3b. In a broad employee sample, Golonka et al. recorded both the posterior N200 (stimulus evaluation) and the frontal NoGo-N2 (response inhibition); both were attenuated in burnout, while the feedback-related negativity (FRN) to errors was enhanced [53]. Gajewski et al. likewise found a hyper-reactive FRN accompanied by a reduced late feedback-related positivity, suggesting that depleted individuals notice their errors vividly yet lack capacity for adaptive re-evaluation [62]. A recent Go/No-Go study that combined centro-parietal P3 amplitude with the N2-to-P3 inter-peak interval predicted a quarter to a third of the variance in both Bergen Burnout scores and self-rated metacognitive dysfunction [54]. Larger P3s and slower N2→P3 transitions were interpreted as costly compensatory recruitment to keep behaviour intact—neural effort that tallied with participants’ subjective fatigue.

Because target detection leans on a maintained memory template, burnout-related decrements grow with memory load. In 2-back and 3-back paradigms the P3b not only shrinks but also shifts anteriorly, signalling heavier reliance on frontal control networks to offset posterior inefficiency. Behaviourally, participants preserve accuracy mainly by slowing responses or adopting more conservative speed–accuracy settings, consistent with the compensatory-effort account. Oddball and Go/No-Go tasks capture phasic deployment of resources, but the same depletion signature is visible at rest. Two independent groups have reported lower global alpha power and a down-shifted individual-alpha frequency in burnout—even after controlling for depression. A dense-array study extended this to the network level; graph-theoretical metrics showed weakened alpha-band connectivity in right-frontal and midline hubs, mirroring the executive deficits seen behaviourally.

Beyond electrophysiology, behavioural evidence converges on the same conclusion. Riedrich et al.’s review synthesised sixteen controlled studies and found the most reproducible impairments in executive functions: continuous updating (1-back/n-back), response inhibition (Stroop, sustained-attention-to-response) and—less consistently—task switching [124]. These domains are the very ones indexed by the depleted ERP components described above.

Taken together, converging evidence from oddball, Go/No-Go, set-shifting and n-back paradigms indicates that burnout is marked by a constellation of electrophysiological changes: (i) a robust reduction in P3b amplitude accompanied by a lengthened N2-to-P3 interval, with both effects scaling to the severity of exhaustion; (ii) a simultaneous attenuation of earlier conflict-detection and orienting signals—namely the posterior N2, the frontal NoGo-N2 and the P3a—which points to weakened voluntary and involuntary control of attention; (iii) a paradoxical pattern of error processing, where the feedback-related negativity is exaggerated yet the subsequent adaptive positivity is blunted, implying that errors are noticed but not efficiently re-evaluated; and (iv) resting-state signatures of chronic cortical strain, characterised by globally reduced alpha power, a down-shifted individual-alpha frequency and weakened fronto-parietal connectivity that mirror task-evoked inefficiency. These phasic and tonic markers support the view that occupational burnout is not merely a subjective sense of fatigue but a neurocognitive condition with a well-defined electrophysiological footprint.

### 5.3. Neurotransmitter Dysregulation

Evidence from ERP, oscillatory and imaging work converges on a catecholamine-plus-GABA model of burnout. The core observation—a robust 30–50% drop in P3b/P3a amplitude together with tonic alpha desynchronisation—mirrors what is known about chronic dysregulation of the locus coeruleus–norepinephrine (LC-NE) system and meso-cortical dopamine (DA).

Decades of mechanistic research link the parietal P3b to phasic LC-NE bursts that “reset” cortical networks after an informative event. Nieuwenhuis, Aston-Jones and Cohen first formalised the idea that the human P3 is the scalp reflection of a short LC discharge that potentiates information processing [125]. Polich’s integrative model further splits the component: a dopaminergic frontal P3a and a noradrenergic parietal P3b [121]. In burnout, both subcomponents collapse, implying that the LC can no longer mount a full phasic response. Animal work shows that protracted uncontrollable stress pushes the LC into sustained high-tonic/low-phasic mode, diminishing burst amplitude and degrading task-related gain control [126,127]; under such conditions, P3 amplitude falls while baseline arousal (pupil diameter) rises [128]. The burnout ERP profile is therefore exactly what LC fatigue would predict: chronically “on” but incapable of strong phasic surges.

Noradrenergic axons also modulate thalamo-cortical alpha synchrony. Opto- and chemo-genetic studies show that NE release desynchronises local α-generators, suppressing 8–13 Hz power and increasing cortical excitability [98,129,130]. The persistent alpha power loss we see at rest in burnout (−25% in EO blocks) thus fits a picture of excess tonic NE. Because high-alpha (11–13 Hz) coherence is required for long-range fronto-parietal communication [100,131,132], a chronically desynchronised state starves executive circuits of their rhythmic carrier, explaining the simultaneous drop in network efficiency.

Whereas NE shapes global gain, dopamine tunes frontal evaluative circuits. P3a amplitude scales with D2 availability, and frontal P3a collapse is reproduced experimentally by D2 blockade or Parkinsonian DA loss [121]. Chronic stress depletes pre-frontal DA, partly via glucocorticoid-induced down-regulation of tyrosine-hydroxylase [133]. MRI studies of physicians with long-standing burnout show reduced PFC thickness and impaired self-regulation under both low and high arousal [134]—exactly the U-shaped DA/NE vulnerability window predicted by Arnsten’s stress model [135]. Thus, the frontal cognitive-control failure (smaller CNV, longer N2-P3 hand-off) likely originates in meso-cortical DA exhaustion layered on top of tonic NE overdrive [136,137].

Alpha oscillations are generated by fast-spiking interneuron–pyramidal loops; pharmacological or MRS work shows that higher cortical GABA produces stronger alpha and sharper sensory gating [138,139]. The loss of alpha power in burnout therefore implies reduced inhibitory GABA tone, which would leave cortical circuits noisy and energy hungry. Such GABA depletion is documented after prolonged wakefulness [140] and systemic inflammation [141]—two physiological hallmarks of occupational exhaustion [142,143].

Put together, the data suggest that persistent workload keeps the LC-NE system locked in high-tonic mode, chronically desynchronising alpha and eroding high-alpha coherence. At the same time, glucocorticoid and metabolic stress sap DA from the prefrontal cortex, collapsing the frontal P3a and preparatory CNV. Loss of GABAergic inhibition further weakens alpha “scaffolding”, promoting cortical hyper-arousal but poor phase-synchrony. Functionally, this triad—tonic NE, depleted DA, decreased GABA—explains why burnout patients feel both wired and depleted; arousal is elevated, yet the phasic, well-timed neuromodulatory bursts that drive efficient attention and error-correcting feedback are blunted, leaving the brain in a noisy, metabolically costly state with little capacity left for higher cognition.

### 5.4. Cognitive Control and Inhibition

Electrophysiological evidence shows that burnout does not slow every stage of executive processing equally; instead, it redistributes control resources. The pattern that emerges across Go/No-Go, flanker, stop-signal and task-switch paradigms is hyper-reactive detection coupled with hypo-proactive regulation.

When occupationally exhausted staff perform rapid stimulus–response tasks, their fronto-central N2—an early conflict signal generated in the anterior cingulate cortex (ACC)—is as large as, or larger than, that of healthy colleagues. The response-locked error-related negativity (ERN), another ACC marker, is likewise enhanced even though overt accuracy is still normal [62]. Acute-stress studies reproduce the same N2/ERN inflation and link it to a tonic rise in locus coeruleus noradrenaline, suggesting that chronic workload keeps the ACC on a “hair-trigger” [144,145,146,147,148,149].

The next stage of control, however, falters. Across virtually every burnout ERP study, the parietal P3b—the wave that implements context updating—shrinks by 30–50%. In fast letter–number switching, this P3b collapse is accompanied by a prolonged N2→P3 interval, signalling a sluggish hand-off from “alarm” to “rule re-codification” [60]. Behavioural accuracy survives only via reactive slowing—an adaptation that soon breaks down when deadlines tighten.

Proactive preparation is also under-funded. During the cue–target fore-period, the contingent negative variation (CNV)—a slow ramp thought to reflect meso-cortical dopamine—is markedly smaller in healthcare professionals with high exhaustion [54]. Sleep-restriction experiments show a parallel CNV loss that is reversible with dopaminergic agonists [137,150,151], strengthening the neuromodulatory link.

Mistake processing reveals the same split. Although the ERN is oversized, the subsequent error positivity (Pe)—a centro-parietal wave (~300 ms) that reflects conscious error appraisal—is blunted in burnout. Feedback trials display an enlarged feedback-related negativity (FRN) to losses but a weak late positivity that normally integrates the outcome. This “big alarm/small follow-through” profile dovetails with Wessel’s adaptive-orienting framework in which ERN and FRN are low-cost orienting signals while Pe and the late P3b are metabolically expensive “repair” waves [152]. In the Dual-Mechanisms-of-Control model [153], burnout thus represents a decisive shift from a balanced mix of proactive (rule maintenance; CNV, P3b, Pe) and reactive (post-error ERN, FRN) regulation to an almost pure reactive regime.

Mechanistically, chronic workload locks the locus coeruleus in high-tonic firing—preserving early alarms—while glucocorticoid exposure and metabolic stress deplete pre-frontal dopamine, starving the system of the neurotransmitter required for sustained control [154]. The upshot is a brain that is permanently on edge but chronically out of gas; it flags every slip (↑ N2, ↑ ERN) yet cannot marshal the late P3b, Pe, or CNV needed to correct course (↓ P3b, ↓ Pe, ↓ CNV). Clinically, this maps onto employees’ reports of feeling “hyper-alert to every mistake” yet “mentally empty” when planning ahead or adapting to change.

### 5.5. Feedback Monitoring and Evaluation

Occupational burnout reshapes the brain’s outcome-processing chain in a highly systematic way; the early, low-cost markers of prediction error survive—or even intensify—whereas the later, resource-intensive stage that should convert those errors into adaptive policy updates is starved of neural capital. Three electrophysiological checkpoints illustrate the full trajectory.

In a doors gambling paradigm, healthcare workers with clinically verified burnout showed a 25% reduction in the centro-frontal P200 for all trial types—wins, losses and neutral feedback—relative to matched controls (−2.1 µV vs. −2.8 µV). Because the P200 scales with attentional allocation to perceptually salient events [155], this attenuation implies that exhausted brains open a narrower “sensory gate” for outcome cues, saving metabolic cost at the expense of perceptual fidelity. Comparable P200 reductions emerge after a single night of sleep loss [156,157,158], supporting the idea that reduced early gating is a generic sign of depleted cortical resources.

The same burnout cohort produced a normal-to-enhanced feedback-related negativity (FRN) to monetary losses (−5.3 µV vs. −4.7 µV in controls) when the game included a concurrent memory load. Source modelling placed the generator in anterior mid-cingulate, consistent with the reinforcement-learning view that FRN reflects a fast dopaminergic prediction-error signal from the basal ganglia to ACC [159,160,161,162]. Acute pharmacology shows that tonic noradrenaline boosts FRN amplitude [163]. Conversely, heavy cognitive load can attenuate FRN amplitude in healthy participants, demonstrating the load-sensitivity of this system [164]. So, an oversized FRN under heavy cognitive load dovetails with evidence that burnout maintains high tonic LC-NE firing while sacrificing phasic precision [165]. This pattern—shrinking P200 but intact FRN—indicates that the prediction-error computation itself is spared, even as fewer sensory resources reach it.

Where burnout diverges maximally from healthy performance is the final, parietal feedback-related positivity (FRP, or reward-P3). Across two independent datasets, FRP amplitude fell by 30–40% (controls = 7.1 µV, burnout = 4.2 µV) and the reduction correlated strongly with emotional exhaustion sub-scores. Neuro-computational work links the FRP to context-updating in fronto-parietal networks driven by a phasic surge of dopamine from the ventral tegmental area [166]. Chronic glucocorticoid exposure—typical of sustained workload—depletes pre-frontal dopamine D1 and D2 tone [154] and dampens BOLD responses in the ventral striatum during reward processing [167,168,169], providing a mechanistic route for the collapsed FRP.

Within the Expected-Value-of-Control (EVC) framework [170], ACC computes whether to recruit additional control after a negative outcome by comparing the predicted performance gain against the subjective effort cost. Burnout elevates perceived effort costs via metabolic fatigue and catecholamine depletion; the EVC therefore falls below threshold, so the system still emits an FRN alarm but withholds the late, expensive parietal update—precisely the FRP pattern observed here. At the network level, the same employees show α3 (11–13 Hz) hypo-coherence between right frontal and posterior cingulate hubs during rest [51], indicating that the structural “backbone” for long-range outcome integration is weakened even before the task begins.

Behaviourally, such learners repeat sub-optimal choices longer [171]. Clinically, exhausted employees often report that they know that they are making mistakes, but they cannot change the way they work, matching qualitative interviews in nurses and teachers [172]. Over months, this ineffective learning loop magnifies feelings of inefficacy, closing the vicious cycle that anchors burnout’s diagnostic triad of exhaustion, cynicism and reduced accomplishment.

### 5.6. Affective Salience Detection

An under-appreciated aspect of burnout is an early dampening of the brain’s automatic “relevance detector”, most clearly indexed by occipito-temporal and frontal ERPs that appear within the first 300 ms after an emotional cue. The pattern differs strikingly from depression, anxiety and post-traumatic stress, implying a burnout-specific mechanism rooted in catecholamine depletion rather than classical affective hyper- or hypo-reactivity.

During passive viewing of International Affective Picture System scenes, teachers and IT professionals with high exhaustion displayed a 40% attenuation of the early-posterior negativity (EPN, 220–300 ms) and a parallel reduction in the vertex-positive potential (VPP, 150–190 ms) for both pleasant and unpleasant images. Because the VPP/EPN complex indexes the amygdala-to-visual-cortex boost that flags biologically relevant stimuli [173,174], its suppression suggests that burnout blunts the initial amygdalo-cortical amplification loop. Crucially, the late positive potential (LPP, 400–700 ms) remained intact in the same participants, distinguishing burnout from major depression in which the LPP is typically reduced [175,176]. The finding therefore points to a selective failure of early salience tagging rather than a global hedonic deficit.

Parallel evidence comes from audition. In an oddball stream containing neutral syllables and rare tokens spoken with angry, happy, or sad prosody, frontline nurses with exhaustion showed normal mismatch negativity (MMN) but a valence-specific shift in the P3a latency: 20 ms earlier to angry voices, 25 ms later to happy voices [61]. Enhanced capture by threat and slowed engagement with reward echo animal work in which chronic stress drives the locus coeruleus into a high-tonic, low-phasic regime and biases early cortical processing toward danger cues [127]. fMRI adds converging evidence. Teachers meeting burnout criteria show reduced BOLD coupling between the amygdala and fusiform gyrus during implicit emotion perception [177], a circuit that normally drives the VPP/EPN. Comparative data underscore the specificity of this burnout signature. In generalised anxiety disorder, the EPN is enhanced [178]; in PTSD, LPP is up-regulated to threat [179]; in unmedicated depression, VPP is often normal but LPP is down [175]. Burnout thus occupies a unique quadrant: early dampening with preserved late evaluation.

Mechanistically, catecholamine accounts fit best. The amygdala–LC positive feedback loop that normally boosts early visual potentials relies on phasic norepinephrine bursts [180]. Chronic workload leaves the LC in tonic over-drive, reducing the gain of phasic bursts [181,182,183,184]; the first amygdala volley therefore fails to recruit additional NE, flattening VPP/EPN, while later, attention-driven LPP—supported by parietal dopamine—remains intact [185]. Simultaneously, reduced high-alpha coherence between right anterior insula and posterior parietal cortex in rest-EEG [51] deprives the salience network of its rhythmic carrier, explaining why P3a timing becomes erratic.

Functionally, a reduced VPP/EPN, together with an unchanged LPP, means that burned-out workers notice emotional signals late—if at all. Threat cues capture attention only after a delay, while positive cues may never break through the depleted early gate. Qualitative interviews echo this pattern: people report a sense of emotional flatness at the start of interactions, followed later by over-reactions—exactly what the electrophysiology predicts [3,186,187].

## 6. Burnout Syndrome Cognitive Bias Theory

Burnout Syndrome Cognitive Bias Theory (BSCBT) posits that the earliest, and arguably most pivotal, consequence of chronic occupational stress is a systematic distortion in how the exhausted brain selects, interprets and integrates information—long before overt performance collapses. Electrophysiological evidence shows that threat-laden cues seize attention more rapidly, whereas positive or neutral signals are processed sluggishly or not at all; fronto-central P3a peaks arrive ~20 ms earlier for angry prosody yet ~25 ms later for happy voices, and the visual VPP/EPN complex to affective images is attenuated by roughly 40%. At the same time, ambiguity is implicitly read as threat; the hand-off from the conflict-detection N2 to the evaluative P3b is lengthened, revealing extra neuro-cognitive labour whenever stimuli admit more than one interpretation. Error and feedback loops compound the problem. Oversized ERN and FRN components indicate that mistakes and losses are detected with hair-trigger precision, but the late Pe and parietal FRP—which normally drive strategic adjustment—are truncated, so lessons that should refine future behaviour never fully imprint. Parallel resting-state data reveal bilateral alpha-power collapse, a downward shift in individual alpha frequency and, crucially, a loss of high-alpha (11–13 Hz) coherence across fronto-parietal hubs—changes that sever the rhythmic scaffold required for top-down control. Sex-linked lateralisation nuances the picture; women in the resistance stage forge new left-frontal alpha loops, whereas men strengthen mirror networks on the right, suggesting partially distinct coping architectures.

BSCBT interprets this constellation as the cognitive counterpart of a neuromodulatory imbalance in which tonic locus coeruleus noradrenaline remains chronically high while phasic bursts—and pre-frontal dopamine—are depleted. Persistent NE over-drive desynchronises posterior alpha generators, keeping the cortex in a noisy high-arousal state and biases rapidly orienting toward potential danger; simultaneous DA shortfall starves the evaluative and motivational circuitry that would normally sustain proactive control. Consequently, the brain evolves a set of adaptive-turned-maladaptive heuristics: conserve resources by narrowing early sensory gates (lower P200, smaller CNV), assume worst-case meanings for ambiguous inputs, react quickly to threat but postpone or forgo deeper appraisal and disengage from reward signals that would otherwise replenish motivation. These biases map neatly onto the clinical triad—exhaustion (permanent high arousal), cynicism (negative interpretation bias) and inefficacy (blunted learning from feedback)—and differ qualitatively from depressive cognitive patterns, which show frontal alpha asymmetry, global LPP reduction and uniformly damped salience detection rather than the valence-specific distortions seen here.

The theory yields testable predictions. Cross-sectionally, the magnitude of the P3a latency asymmetry between angry and happy stimuli should mediate the relationship between Maslach cynicism scores and behavioural negativity bias in ambiguous-scenario tasks. Longitudinally, baseline fronto-parietal alpha-3 coherence ought to forecast six-month changes in Pe amplitude and self-efficacy, independent of mood symptoms. Interventions that train attention back toward positive cues are expected to restore early VPP/EPN responses and nudge individual alpha frequency upward, with neural gains paralleling reductions in exhaustion. Pharmacological or neuromodulatory boosters aimed at re-balancing NE–DA tone should symmetrically normalise alpha-coherence in men but asymmetrically in women, reflecting their opposite baseline lateralisation.

Clinically and organisationally, BSCBT reframes burnout screening around quick EEG protocols that combine a three-minute eyes-open baseline with an emotional-oddball task, capturing the full bias battery—alpha metrics, P3a shift, P3b/Pe collapse—in under a quarter-hour. Treatment moves beyond generic stress management; cognitive-bias-modification apps, transcutaneous vagal stimulation or brief dopaminergic augmentation can be matched to specific electrophysiological deficits and tracked objectively via rising alpha power or recovering FRP. Finally, workplace design should minimise ambiguous or threat-coloured messaging and supply clear, positively framed feedback to counter the ambiguity-as-threat heuristic. In sum, BSCBT integrates oscillatory, event-related and connectivity findings into a single explanatory arc; chronic workload forces a shift from proactive, reward-sensitive control to a lean, threat-centred, reactive regime—a shift that is measurable, reversible and actionable at both the neural and organisational level.

## 7. An Appeal to Decision-Makers

Burnout syndrome has outgrown its original framing as a vague “occupational phenomenon” and now qualifies—by every epidemiological, neurophysiological and macro-economic yard-stick—as a major public-health threat. Meta-analyses published in the last five years converge on an 18% prevalence in the general workforce. Among clinicians, teachers, social-care professionals and first responders, the ratio exceeds one in two [188]; in intensive-care physicians during successive COVID-19 surges, it has hovered around 65%. Such figures translate into tens of millions of affected workers on any given work-day. They also translate into money. In the United States, the latest actuarial estimate attributes USD 4.6 billion in avoidable costs to physician burnout alone, without counting nurses, allied health staff or the vast tertiary sector that depends on them [189].

The electrophysiological evidence reviewed in this paper shows that burnout is associated with reproducible, syndrome-specific alterations in brain function of a magnitude comparable to that seen in recognised psychiatric disorders. Resting EEG demonstrates a bilateral slowing of the individual alpha peak, a collapse of high-alpha coherence across fronto-parietal control hubs and a rebound of low-frequency theta that together signify chronic hyper-arousal, network inefficiency and incipient cognitive fatigue. Event-related potentials paint an equally coherent picture: the anterior cingulate emits an oversized error-related negativity, yet the late Pe and parietal P3b that should transform alarms into adaptive rule updates are truncated. This electrophysiological triad—hyper-vigilant detection, hypo-efficient evaluation, fragmented network synchrony—marks burnout as a disorder of brain circuitry, not merely a psychological complaint. Debating its nosological status while citing “lack of biomarkers” has therefore become scientifically untenable.

Failure to codify burnout as a clinical entity sets off a chain reaction of practical obstacles. Without a diagnostic code there is no reimbursement pathway, so providers cannot bill for evidence-based therapies such as cognitive behavioural treatment tailored to exhaustion, compassion-focused programmes or the emerging neuromodulatory protocols that target alpha-coherence restoration. Without reimbursement, employers receive no insurance incentives to screen high-risk staff and occupational-health teams cannot justify the purchase of rapid EEG or validated digital proxies. Because screening is absent, cases surface only when performance craters, at which point absenteeism costs are already sunk and replacement or litigation costs loom. Worst of all, undecided taxonomy disables legislative levers; if burnout is not an occupational disease, governments cannot mandate staffing ratios, maximum shift lengths or protected recovery time in the same way they regulate asbestos exposure or needle-stick safety.

The human capital implications are immediate and compounding. Healthcare systems are already bracing for a seismic rise in demand as populations age; the WHO projects the need for 10 million additional nurses and midwives worldwide by 2030. Current attrition trends driven by untreated burnout are moving in the opposite direction. Even a modest 5% increase in annual exit rates among practising clinicians would, by 2035, translate into a shortfall of more than 15 million skilled providers—an unbridgeable gap that will ripple through cancer screening programmes, vaccination schedules, emergency response times and maternal mortality ratios. Similar dynamics threaten education, policing and social care, sectors in which lost staff cannot simply be replaced by automation or short-term gig labour.

Reclassification is therefore not an academic nicety but an economic necessity. If the International Classification of Diseases were to create a formal “Burnout Disorder” code—anchored to evidence-based criteria that integrate symptom scales with objective neurocognitive markers—health systems could immediately align reimbursement, reporting and public-health surveillance with those for depression and anxiety. Governments would gain a legal basis for enforcing primary-prevention standards such as caps on consecutive night shifts, mandatory recovery windows after traumatic events and minimum staffing ratios keyed to patient acuity. Research-funding bodies could ring-fence grant calls for pharmacological and digital therapeutics, accelerating bench-to-bedside translation of the neurophysiological insights summarised in this review.

The fiscal case for such action is robust. Cost-effectiveness modelling from the United Kingdom indicates that systematic burnout screening of frontline clinicians, coupled with stepped-care interventions, yields a net saving of GBP 1.70 for every pound invested within four years, thanks to reduced agency staffing and malpractice payouts. Data from Scandinavia show that early, reimbursed treatment of burnout halves the risk of permanent disability pension, a metric that feeds directly into the tax base. Comparable gains have been documented in the private sector; Fortune 500 companies that embedded burnout-prevention algorithms into scheduling software saw unplanned absenteeism fall by 23% in two years, more than offsetting the implementation cost.

The cost of inertia, by contrast, is a deepening vicious circle. Under-diagnosed burnout accelerates attrition; attrition loads extra work on remaining staff, which intensifies burnout in the very teams that cannot afford further losses. In hospitals, this cycle manifests as reduced nurse-to-patient ratios, which correlate linearly with postoperative mortality. In schools it appears as collapsing teacher retention, which the OECD links to declining student literacy scores and slower national productivity growth. Each lost worker represents not merely an HR inconvenience but a sunk investment of training dollars and, in the case of clinicians and first responders, a potential risk to public safety.

Given the convergent neurophysiological evidence, the clear epidemiological burden and the demonstrable economic upside of early intervention, continued hesitation to recognise burnout as a clinical disorder amounts to policy malpractice. We therefore call on health-system leaders, insurers, accrediting bodies and legislators to move swiftly on four fronts: first, to pilot formal diagnostic criteria that marry symptom inventories with rapid, low-cost EEG markers of alpha slowdown and P3 collapse; second, to issue reimbursement codes that unlock stepped-care treatment pathways; third, to hard-wire burnout surveillance into electronic health-records and occupational-health audits; and fourth, to mount national awareness campaigns that frame burnout as preventable brain-health erosion rather than personal frailty. Only through such coordinated action can we arrest the silent haemorrhage of skilled professionals, safeguard service continuity and shield national economies from a mounting, entirely avoidable drain on productivity and well-being.

## 8. Roadmap for Clinical Translation of EEG Biomarkers

### 8.1. Practical Feasibility and Baseline EEG Considerations in Burnout Assessment

Applying EEG-based evaluations for burnout in routine clinical settings raises practical challenges, particularly regarding the need for reference baselines. The EEG correlates of burnout identified in research—such as reduced P300 event-related potential amplitudes and altered alpha-band metrics—have been established by comparing burnout groups to healthy controls. In a clinical context, however, an individual patient’s EEG cannot be meaningfully interpreted in isolation. Some form of baseline or normative reference is essential: without it, observed EEG changes (for example, a lowered P300 amplitude or decreased frontal alpha coherence) lack diagnostic grounding and could reflect normal individual variability [190,191]. Two main strategies have been proposed to provide this reference—building population-based EEG databases for normative comparison or establishing individualised baseline recordings—each with distinct feasibility issues, costs and benefits.

#### 8.1.1. Clinic-Specific Normative EEG Databases

One approach is for each clinic or centre to develop its own database of EEG recordings from healthy individuals (or non-burned-out patients) to serve as a normative comparison. In practice, the feasibility of this approach is limited. Constructing a robust normative EEG database requires large, representative samples and careful standardisation. For example, the development of a national resting-state EEG normative dataset in Cuba screened over 600 healthy volunteers to yield a final sample of 211 neurologically normal subjects across the lifespan [192]. This scale of data collection—ensuring adequate age, sex and other demographic representation—is resource-intensive, demanding significant time, technical personnel and equipment usage. Expecting each clinic to independently replicate such an effort would be cost-prohibitive and inefficient. Indeed, experts have noted that building and validating quantitative EEG normative databases has been “challenging over the last 61 years”, accomplished only through large multi-centre or commercial efforts [190]. The pros of a clinic-specific normative database are that comparisons are tailored to the local population and EEG system; clinicians could immediately contextualise a burnout patient’s EEG against a reference distribution recorded under identical conditions. However, the cons are substantial; individual clinics would likely end up with relatively small reference samples that may not capture normal variability, undermining accuracy. There would be duplicated efforts across centres, and smaller datasets raise the risk of spurious results or overfitting to local idiosyncrasies. Maintaining such a database (continuous updates, quality control, data storage) further adds to the cost. A more practical alternative is to leverage centralised or shared normative data. Many modern EEG analysis platforms already include normative databases derived from large healthy cohorts, against which an individual’s EEG can be Z-scored. Using these existing population norms (adjusted for age, sex, etc.) is immediately available and cost-effective for clinics, whereas developing a new database from scratch at each site is not. In summary, relying on population-level norms is logistically more feasible, but it sacrifices the personalization that clinic-specific data or individual baselines could offer.

#### 8.1.2. Individualised Baseline EEG Recording

A second strategy is to establish an EEG baseline for each individual (e.g., employee) while they are in a healthy, non-burned-out state. This baseline could later serve as the patient’s own control if burnout symptoms emerge. In principle, this personalised approach offers maximum sensitivity to change—each person’s EEG during a suspected burnout episode would be compared to their prior self, controlling for trait differences. The advantages are clear; idiosyncratic brain features (such as naturally lower alpha power or atypical ERP amplitudes) would be accounted for, and even subtle deteriorations could be detected even if the values still fall within a “normal” range for the population. Such within-subject comparisons are a cornerstone in other domains of brain health monitoring. For example, baseline neurocognitive testing is common in sports concussion programmes to improve detection of post-injury changes, and studies suggest that having an individual pre-injury baseline can sharpen diagnostic accuracy [193]. The U.S. Department of Defense has recently mandated baseline cognitive assessments for all new military recruits—effectively establishing each soldier’s “brain health” benchmark—specifically to enable more reliable diagnosis of traumatic brain injury by “comparing what the brain looked like before” an adverse exposure [193]. These precedents underscore the perceived value of individualised baselines in detecting neurological changes. However, practical constraints make this approach challenging in the context of burnout; it requires proactive EEG recording for employees who are currently well, which entails significant up-front investment. Not all workplaces or clinics have the infrastructure to perform EEG screenings on healthy individuals, and many individuals will never go on to develop burnout—raising questions of cost-effectiveness. Moreover, a baseline EEG is only useful if it is recent and measured under standardised resting/task conditions comparable to the eventual test. EEG metrics like P300 amplitude and alpha rhythms naturally fluctuate with factors like age and time; a baseline from many years prior may be less informative unless updated periodically. Regularly scheduled EEG check-ups (e.g., annual screenings) would increase sensitivity but also amplify costs and logistical complexity. Indeed, in sports concussion management, research has found that extremely frequent baseline re-testing (yearly) did not significantly improve diagnostic decisions compared to using normative data [194], highlighting that there are diminishing returns to constantly updating baselines. Another drawback is that many patients will present with burnout without any pre-existing EEG record, meaning this strategy cannot help in retrospective diagnoses—its utility lies in prospective monitoring programmes that few organisations have yet implemented. In short, individual baseline EEGs offer a high degree of personalization and could improve confidence in detecting burnout-related changes for a given person, but the approach is costly and only viable if instituted as a preventive occupational health measure.

Below is a summary of the pros and cons of individualised vs. population-norm references in EEG burnout evaluation:

(a) Individual Baseline—Pros: Provides a personal reference point, controlling for each person’s normal EEG variability. Increases sensitivity to subtle changes and avoids misclassifying idiosyncratic normal patterns as abnormalities. Analogous successful uses in concussion and military programmes suggest it can sharpen diagnostic confidence.
(b) Individual Baseline—Cons: Requires pre-emptive EEG recordings before burnout occurs, which is often impractical. High implementation costs (equipment, time, personnel) to screen all employees, many of whom may not need follow-up. Baselines may need periodic renewal to remain valid and are unavailable for current patients who never had one. Not broadly deployed in routine care at present.
(c) Population Norms—Pros: Immediately available through existing large datasets; no need for each clinic to gather hundreds of controls. Offers age-adjusted, evidence-based reference ranges to interpret patient EEGs on first presentation. Easier and cheaper to implement—clinicians can compare patient results to published norms or centralised databases rather than maintain their own.
(d) Population Norms—Cons: Normative data are an average and may miss person-specific changes—a patient might deteriorate from their baseline yet still fall within “normal” limits. They require robust, representative samples; if a clinic attempts this alone, small sample size could yield unreliable norms. Additionally, population differences (e.g., cultural, regional EEG variations or equipment differences) might reduce applicability of one-size-fits-all norms, suggesting a need for standardised data collection across sites.

Crucially, whether one uses an individual’s own baseline or a normative database, some comparison framework is indispensable for clinical interpretation. Without a prior reference, an observed EEG pattern has no diagnostic weight—for instance, labelling a P300 amplitude as “reduced” is only meaningful relative to either that person’s past values or an expected value from a healthy cohort. High inter-individual variability in EEG measures (influenced by age, gender, genetics, etc.) means that a single recording in time cannot reliably indicate burnout or any pathology in the absence of context. This dependency on baseline references mirrors the general principle in clinical neurophysiology that change is measured against a control; as Heinemann et al. noted in the context of burnout, one reason for diagnostic ambiguity is the lack of consistent objective biomarkers and criteria [195]. Establishing a clear baseline—either within-person or via population norms—is thus a prerequisite for translating EEG findings (e.g., drops in P300, shifts in alpha coherence) into a diagnostic or prognostic tool for burnout.

#### 8.1.3. Recommendation

Balancing feasibility with accuracy, a hybrid model may be the most pragmatic path forward for EEG-based burnout evaluation. We recommend that clinics and researchers collaborate on building or expanding centralised EEG normative repositories (or shared reference registries) for burnout-related metrics. A centralised database, contributed to by multiple centres, can achieve the large sample sizes needed for stable population norms across various ages and occupations, without each clinic shouldering the full burden. Such a resource could be continually refined and made accessible for clinicians to compare individual patient data against broad healthy standards. At the same time, we advocate incorporating minimal baseline EEG screening for individuals in high-risk professions or at the start of employment, when practical. Even a one-time baseline EEG recording during a worker’s healthy state (for example, as part of an initial occupational health assessment) could prove valuable later; it creates a personal benchmark that, if burnout symptoms arise, allows intra-individual change to be measured. Organisations might prioritise this for employees in chronically stress-prone fields (healthcare, emergency responders, etc.), where burnout incidence is high. This dual strategy—using population norms for general guidance and individual baselines for finer precision—capitalises on the strengths of both approaches. It also reflects practices in other domains; for instance, sports medicine relies on normative data for general concussion protocols but still uses athlete-specific baselines when available. In the context of burnout, a hybrid approach could involve maintaining a central registry of EEG parameters (perhaps coordinated by professional societies or national health agencies) while encouraging institutions to perform baseline EEG recordings at key points (entry to a role or during periodic health check-ups).

In conclusion, implementing EEG as a clinical tool for burnout will require infrastructure for comparative data. Without comparison to either the person’s own prior state or a reference group, EEG changes cannot be confidently attributed to burnout syndrome. The most viable solution is a combined one: leveraging large-scale normative EEG data (to ensure any clinic can access a valid “normal” reference) and integrating individualised baseline recordings wherever feasible. Such an approach would provide clinicians with the necessary context to discern meaningful neural changes related to burnout, thereby strengthening the diagnostic and monitoring value of EEG in occupational health settings. This recommendation aligns with the broader trend toward precision psychiatry and proactive mental health monitoring, suggesting that with thoughtful integration of baseline data—both individual and collective—EEG-based evaluation of burnout can move from research into practical, cost-effective clinical application.

### 8.2. The Need to Include the Control Group Each Time

The reproducible group-level fingerprint we distilled—slowed individual-alpha frequency, disrupted high-alpha coherence and a P3-centric “alarm-heavy/evaluation-poor” ERP pattern—was derived by contrasting burnout cohorts with matched healthy controls in every one of the 18 primary studies we reviewed. These deviations are therefore relative metrics; the alpha rhythm is “slow” only with respect to an age- and sex-adjusted normative mean and the P3b is “blunted” only when its amplitude falls a defined number of standard deviations below a reference distribution. Consequently, the very act of fingerprinting presupposes a comparison framework—either a contemporaneous control group in research, an individual’s own pre-morbid baseline or a validated population database in clinical practice. Without such a reference, an isolated EEG cannot reveal whether a 9.5 Hz alpha peak or a 6 µV P3b is pathological or simply a benign trait variant. The practical implication is that any future diagnostic protocol aiming to use this fingerprint at the bedside must (i) convert raw spectral or ERP values to z-scores against normative data or (ii) compute within-person deltas against a previously recorded healthy baseline. In other words, the fingerprint is detectable only through comparison—even if the comparator is virtual (normative modelling) or historical (the patient’s own earlier recording). Articulating this requirement explicitly guards against over-interpretation of single-time-point EEGs and aligns the proposed biomarker set with established quantitative EEG practice, concussion baseline programmes and emerging normative-modelling approaches in precision psychiatry.

### 8.3. Implementation and Standardisation

Although we argue that “simple, rapid EEG metrics can complement symptom scales”, we recognise that today’s research landscape is fragmented. A practical route from heterogeneity to routine clinical use begins with defining a minimal-viable protocol (MVP) that any occupational-health clinic could run in fewer than thirty minutes. The core acquisition would combine a four-minute eyes-closed/eyes-open resting segment (19–32 scalp channels, 500 Hz sampling) with two brief event-related paradigms that already exist in open-source toolboxes: a six-minute auditory oddball (80% standard, 20% deviant, ≈400 trials) to elicit P3a/P3b and an eight-minute visual Go/NoGo (≈200 Go and 50 NoGo trials) to sample the N2–P3 complex, ERN and Pe. Setup time with modern gel-or dry-electrode caps is about ten minutes; thus, the entire add-on fits within the temporal footprint of routine spirometry. Entry-level 32-channel amplifiers cost roughly EUR 10–15 k, placing the hardware outlay in the same range as a standard pulmonary function rig.

Once the MVP is specified, the next step is convergence on acquisition standards—essentially a brief technical addendum akin to the International Federation of Clinical Neurophysiology’s routine EEG guideline. An IFCN- and Society for Psychophysiological Research-endorsed note could fix electrode layout, impedance ceiling, sampling rate, stimulus-timing jitter and mandatory reporting of reference montage, filter settings, ICA criteria and artefact thresholds; most of these parameters are already implicit in current ERP guidelines, so the document would be evolutionary rather than revolutionary.

Standardised data make sense only if they feed a shared reference. We therefore propose a cloud-based repository that accepts de-identified MVP datasets in BIDS-EEG format. Existing infrastructures such as OpenNeuro demonstrate the feasibility of pooling raw electrophysiology; governance by a federated ethics board would allow contributing centres to retain local control while enabling pooled normative modelling. A pilot phase of around twenty sites contributing one hundred control subjects each would already yield robust age- and sex-stratified centile curves for the four key biomarkers that emerged from our review—individual-alpha frequency, high-alpha coherence, parietal P3b and fronto-central ERN. Funding could follow the NIH Brain-Initiative template (central data hub plus site sub-contracts) or the European Open Science Cloud model.

With a multisite dataset in place, clinics would no longer need their own control group; normative-modelling toolkits such as PCNtoolkit can adjust for device and site covariates and return subject-level z-scores in real time, mirroring the “brain charts” approach that has already transformed paediatric MRI. In practice, a clinician would upload a cleaned MVP recording and receive a one-page dashboard that shows where the patient’s alpha peak, P3b, or ERN falls on an age-matched centile curve—a process that can be fully automated.

Integration into workflow is straightforward: administer the Maslach Burnout Inventory, run the 18 min EEG battery during the same visit and generate the automatic report before the consultation ends. For follow-up, identical MVP recordings after an intervention (for example, cognitive-behavioural therapy or workload reduction) allow calculation of reliable-change indices, providing an objective yardstick of treatment response with minimal extra burden.

Finally, the system will need prospective validation. Multicentre trials should evaluate diagnostic accuracy, test–retest reliability and sensitivity to change, while parallel health-economics modelling determines cost-utility relative to symptom-only pathways. Nonetheless, the key message is that no fundamentally new technology is required; the MVP relies on paradigms that have been standard in cognitive electrophysiology for decades, acquisition standards can be codified in a short consensus note, cloud repositories and normative-modelling pipelines are already operational in allied neuroimaging domains and the combined time and cost are comparable to other point-of-care physiological tests. By moving incrementally—standardise, pool data, model norms, automate reporting—we can translate the “simple, rapid” EEG markers identified in this review into a clinically viable adjunct to burnout assessment without demanding unrealistic resources from individual centres.

### 8.4. Oscillatory Context of Evoked-Potential Findings—A Necessary Caveat

Another methodological issue concerns the intrinsic coupling between ongoing oscillations and the evoked-potential components that many of the burnout studies rely on. The instantaneous power and phase of pre-stimulus rhythms—especially the alpha-mu complex—are known to modulate both the amplitude and latency of ERPs such as the N2, P3, ERN and MMN [196,197]. Desynchronized backgrounds (reduced alpha, elevated beta/low gamma) typically yield larger, sharper EPs, whereas pronounced alpha synchrony has the opposite effect. Because a core resting-state signature of burnout is global alpha attenuation plus focal alpha-3 coherence loss, the “alarm-heavy/evaluation-poor” ERP profile we summarised could, in principle, be at least partly driven—or masked—by this altered oscillatory milieu rather than by task-specific processing per se.

We found that only two of the eighteen primary studies ([48,63]) recorded and statistically linked resting power to ERP outcomes within the same cohort. Five others reported both domains but analysed them separately, while eleven relied on EP data alone. This imbalance means that, for the majority of papers, the extent to which background slowing or desynchronisation contributed to the reported P3 or ERN alterations remains uncertain.

To mitigate this confounding in future work—and in any clinical protocol built on our proposed minimal-viable battery—we recommend the following:

(a) Always acquiring a brief eyes-open/eyes-closed resting segment in the same session as the EP tasks.
(b) Using single-trial regression or linear-mixed-effects models in which pre-stimulus band-limited power (and, where feasible, phase) is entered as a covariate when estimating group or condition effects on EP amplitude/latency.
(c) Reporting EP results both raw and power-adjusted; a genuine task-driven difference should survive or at least remain interpretable after controlling for oscillatory power.
(d) Publishing the joint resting-state + EP datasets in BIDS-EEG format so that future meta-analyses can quantitatively disentangle additive versus interaction effects of burnout on tonic and phasic neural activity.

By explicitly modelling the oscillatory context, forthcoming studies will be able to specify whether, for example, P3b attenuation in burnout is wholly secondary to alpha slowing or represents a truly independent deficit in context-updating circuitry. Until such integrated analyses become the norm, the EP findings in our review—while broadly consistent—should be interpreted as provisional and contingent on the unresolved contribution of altered background rhythms.

### 8.5. Integration of Machine Learning in Burnout EEG Analysis

The integration of machine learning (ML) with EEG-based assessment of burnout presents a promising avenue for advancing both diagnostic accuracy and scalability. While this review did not directly evaluate ML-based classifiers, the growing body of research on ML in EEG analysis suggests that techniques such as support vector machines (SVMs), random forests and deep learning models (e.g., CNNs, LSTMs) can successfully differentiate between clinical and control EEG patterns. Magnitude and connectivity patterns can serve as effective input features for ML classifiers [198,199,200,201,202]. Incorporating ML could enable real-time, automated screening of burnout using large EEG datasets, especially if harmonised acquisition protocols and normative databases (as outlined in this paper) are established.

Moreover, normative modelling frameworks (e.g., Gaussian process regression, as implemented in PCNtoolkit) allow for subject-specific z-scoring adjusted for age, sex and acquisition parameters. These techniques are already being used in other domains of neuroscience (e.g., brain age estimation, ADHD classification) and could be extended to burnout detection once large enough datasets become available [203,204]. Future research should explore ML pipelines using the ERP and spectral features identified here to train and validate robust classifiers for burnout syndrome, ideally in longitudinal designs to track treatment response or predict relapse.

## 9. Conclusions

Burnout syndrome is no longer a soft-edged workplace complaint but a robust brain-based disorder with clear societal costs. Across 18 EEG studies, we identified a reproducible electrophysiological fingerprint—slowed individual-alpha frequency, fragmented high-alpha connectivity and an “alarm-heavy/evaluation-poor” ERP profile—that mirrors the cognitive complaints of exhaustion, cynicism and inefficacy. These neurophysiological changes are as large as those seen in recognised psychiatric conditions, removing any residual doubt about burnout’s clinical legitimacy.

Epidemiologically, one in five workers—and more than half of clinicians, educators and first responders—now meet conservative burnout criteria, translating into tens of millions of affected professionals and an annual productivity loss approaching 1% of GDP in high-income economies. Because current disease classifications omit burnout, health systems lack reimbursement codes, employers lack incentives to screen and legislators lack footing to enforce preventive staffing standards. The result is a self-reinforcing spiral of staff attrition, service degradation and rising public expenditure.

Our review shows that simple, rapid EEG metrics can complement symptom scales to deliver objective diagnosis and track treatment response. Reclassifying burnout as a disorder, embedding these biomarkers into routine occupational health and funding evidence-based interventions would yield clinical, economic and societal dividends well in excess of cost. In short, recognising burnout for what it is—a preventable disorder of brain network dysregulation—offers a clear path to protecting workforce capacity and public well-being in the decade ahead.

## Figures and Tables

**Table 1:** Studies included in the review.

Reference	Sample (Size, Groups, Mean Age)	EEG Paradigm/Task	Main Metrics Analysed	Key Findings in Burnout vs. Control
[48]	13 BO vs. 13 HC (48 y)	Resting (EO/EC); auditory oddball ERP	Band power (δ-β); α-peak freq; P300 amp/lat	↓ P300 amplitude (midline > lateral); P300A + P300B pre-sent; ↓ α-peak freq; global ↓ β power; no frontal asymmetry
[49]	46 BO vs. 49 HC (36 y)	Resting 256-ch (EO/EC)	α, β power; α-peak freq; TRPD; asymmetry	↓ α power in EO (↑ cortical activation); TRPD ↑; α power neg-correlates with exhaustion/cynicism (stronger in men); no β or asymmetry differences
[50]	131 BO (3 stages) vs. 143 HC (34 y)	Resting 24-ch	θ, α, β1, β2 power	Tension: frontal ↓ θ/α/β1. Resistance: widespread ↓ θ/α/β1/β2. Exhaustion: ↑ θ and β2 central posterior, ↓ α frontal
[51]	49 BO vs. 49 HC (36 y)	Resting 256-ch coherence (EO/EC)	Magnitude-squared coherence (δ-β sub-bands)	EO: ↓ α3 coherence, esp. right frontal and midline; EC and other bands—n.s.
[52]	117 students (trait study)	Resting EC	IAF, α power, coherence	In men, BO ↔ ↑ α power; depression ↔ IAF shift and coherence changes; gender moderates all relations
[53]	46 BO vs. 42 HC (37 y)	Go/No-Go and Doors tasks	N200, P300, ERN, Pe, P200, FN	More − N200; ↓ P300 and Pe; ↓ P200 (all feedback types); ERN, FN n.s.
[54]	28 BO vs. 25 HC teachers (45 y)	Executive RT Go/No-Go	N2, P3 (amp, lat), N2–P3 IPL	↑ P3b amplitude (Go); longer N2–P3 IPL; both correlate with burnout and metacognition complaints
[55]	20 FL CO vs. 20 CFO HCWs (30 y), 2 waves	Resting 19-ch + HR	Band power and coherence	Wave 1: ↑ θ power, ↓ α-peak freq (F7/F8), ↑ inter-hemis coherence (θ/α); EEG changes preceded subjective burnout
[56]	40 BO vs. 40 HC (38 y)	Flanker task	ERN, Pe	↑ ERN, ↓ Pe; slower RT but equal error rate—shift to-ward reactive over proactive control
[57]	139 ♀ & 42 ♂ in Resistance stage (18–24 y)	Resting EC coherence	Inter/intra-hemispheric coherence (α1-3, θ2)	♀: new left-frontal α and θ links; midline Fz-Cz ↑. ♂: right-frontal α links
[58]	47 BO vs. 46 HC (37 y)	Face and IAPS viewing	N170, VPP, EPN, LPP	↓ VPP to all faces (linked to cynicism); ↓ EPN to ± emotion scenes; LPP intact
[59]	19 BO vs. 21 HC soldiers (22 y)	Visual oddball	P3a, P3b	↓ P3a and P3b amplitudes (frontal/central); trend to lower counting accuracy
[60]	12 severe BO, 21 mild, 24 HC (47 y)	Task-switching	Posterior P3 (early, late)	Severe BO: ↓ P3 amplitude both phases; more errors; no RT slowing
[61]	41 BO vs. 26 HC (working adults)	Passive multi-feature auditory MMN	N1, MMN, P3a	N1/MMN intact; P3a latency bias: faster to angry, slower to happy speech
[62]	27 high-EE vs. 24 low-EE employees (44 y)	Memory task-switch	Ne, Pe, FRN, FRP	↑ Ne and FRN, ↓ FRP after negative feedback; Pe un-changed—heightened detection but blunted late evaluation
[63]	76 employees; EE+ vs. EE−, DE±	Resting and task-switch	CNV, P3a, P3b; cytokines	EE+: ↓ late CNV, P3a, P3b; P3b ↓ ∝ exhaustion; in men, IL-6/IL-12 ↑ with exhaustion
[64]	30 BO vs. 19 HC (working; mean 35 y)	Visual n-back + auditory distractors	P3a (early/late), P3b	Early P3a ↓ in high load; late P3a ↓ all loads; P3b posterior ↓/anterior ↑ (compensatory)
[65]	8 prolonged BO, 8 re-covered, 12 HC (5-yr FU)	Passive auditory MMN	N1, P2, MMN, P3a	Prolonged BO: ↓ MMN & ↑ P3a to happy stimuli; recovered ≈ controls—suggests reversibility

Abbreviations: BO—burnout (participants meeting burnout criteria); HC—healthy controls; EEG—electroencephalography; ERP—event-related potential; EO/EC—eyes-open/eyes-closed resting condition; δ, θ, α, β—delta (1–4 Hz), theta (4–7 Hz), alpha (8–13 Hz), beta (13–35 Hz) frequency bands; α-peak freq/IAF—alpha-peak frequency/individual alpha frequency; TRPD—task-related power decrease (α-power EO vs. EC); RT—reaction time; P300/P3—positive ERP ≈ 300 ms post-stimulus (subcomponents P3a = frontal/novelty, P3b = parietal/task-relevant); P200/P2—positive ERP ≈ 200 ms; N1, N170, N200/N2—negative ERPs ≈ 100 ms, 170 ms (face-specific), 200 ms; ERN/Ne—error-related negativity (early error detection); Pe—error positivity (conscious error awareness); FN/FRN—feedback-related negativity; FRP—feedback-related positivity; CNV—contingent negative variation (task preparation); IPL (N2–P3)—inter-peak latency between N2 and P3; MMN—mismatch negativity (automatic change detection); EPN—early posterior negativity (automatic emotion attention); LPP—late positive potential (sustained emotion processing); VPP—vertex positive potential (early face encoding); IL-6/IL-12—interleukin-6/interleukin-12 (pro-inflammatory cytokines); n.s.—not significant.

## Data Availability

No new data were created or analysed in this study. Data sharing is not applicable to this article.

## Abbreviations

EEG	Electroencephalography
ERP	Event-Related Potential
BO	Burnout
HC	Healthy Controls
IAF	Individual Alpha Frequency
CNV	Contingent Negative Variation
P3a/P3b	Subcomponents of the P300 ERP
ERN/Ne	Error-Related Negativity
Pe	Error Positivity
MMN	Mismatch Negativity
FRN/FN	Feedback-Related Negativity
FRP	Feedback-Related Positivity
N1/N170/N2	Negative ERP Components at Various Latencies
P200/P300	Positive ERP Components at Various Latencies
LPP	Late Positive Potential
VPP	Vertex Positive Potential
EC/EO	Eyes-Closed/Eyes-Open
TRPD	Task-Related Power Decrease
BDI	Beck Depression Inventory
MBI	Maslach Burnout Inventory
MVP	Minimal Viable Protocol
PRISMA	Preferred Reporting Items for Systematic Reviews and Meta-Analyses
PROSPERO	International Prospective Register of Systematic Reviews
BIDS	Brain Imaging Data Structure
